# Supplementary material for: Evaluating the impact of the membrane thickness on the function of the intramembrane protease GlpG
Source: Biophys J. 2024 Nov 1;123(23):4067–81. doi: 10.1016/j.bpj.2024.10.019 (PMC11628809; doi:10.1016/j.bpj.2024.10.019)
Supplement: Document S1. Figures S1–S8 and Tables S1–S4 [file mmc1.pdf]

Biophysical Journal, Volume *123*

Supplemental information

Evaluating the impact of the membrane thickness on the function of the intramembrane protease GlpG

Oskar Engberg, Anjana V. Mathath, Viola Döbel, Christian Frie, Marius K. Lemberg, Debashree Chakraborty, and Daniel Huster

Supporting Information

Evaluating the Impact of the Membrane Thickness on the Function of the Intramembrane Protease GlpG

Oskar Engberg,[1] Anjana V. Mathath,[2] Viola Döbel,[1] Christian Frie,[3] Marius K. Lemberg,[3] Debashree Chakraborty,[2] Daniel Huster[1,]*

[1]Institute for Medical Physics and Biophysics, University of Leipzig, Härtelstr. 16/18,

D-04107 Leipzig, Germany

[2] Biophysical and Computational Chemistry Laboratory, Department of Chemistry, National Institute of Technology Karnataka, Mangalore, 575025, Karnataka, India

[3]Center for Biochemistry and Cologne Excellence Cluster on Cellular Stress Responses in Aging-Associated Diseases (CECAD), University of Cologne, Joseph-Stelzmann-Str. 52, D-50931 Cologne, Germany

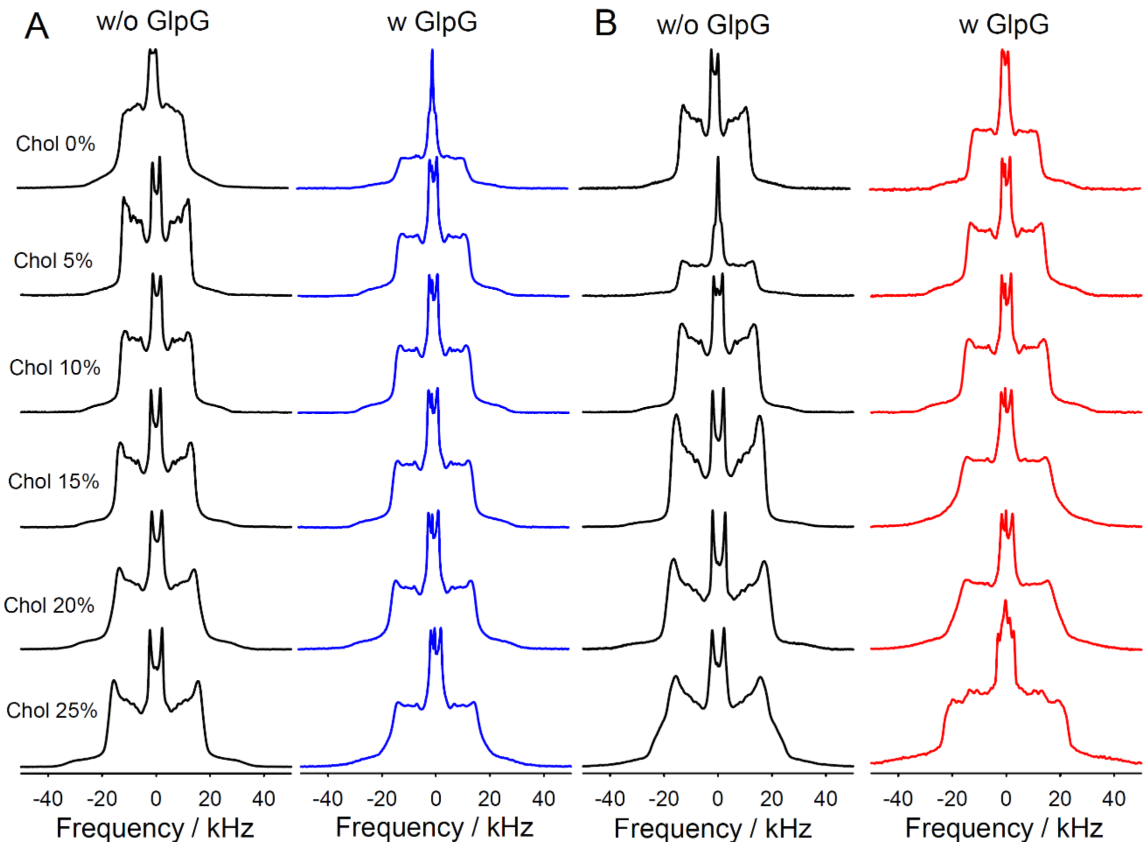

Figure S1. Static ^{2}H NMR spectra of DLPC-d_{46} and DMPC-d_{54} membranes with increasing amount of cholesterol in the presence and absence of GlpG. ^{2}H NMR spectra of DLPC-d_{46} in panel A) and DMPC-d_{54} in panel B. The indicated cholesterol concentrations correspond to the prepared concentrations. Measurements were carried out for MLVs hydrated with excess acetate buffer at pH 4 and a temperature of 37°C. The cholesterol-free spectra are reproduced from (17) with permission.

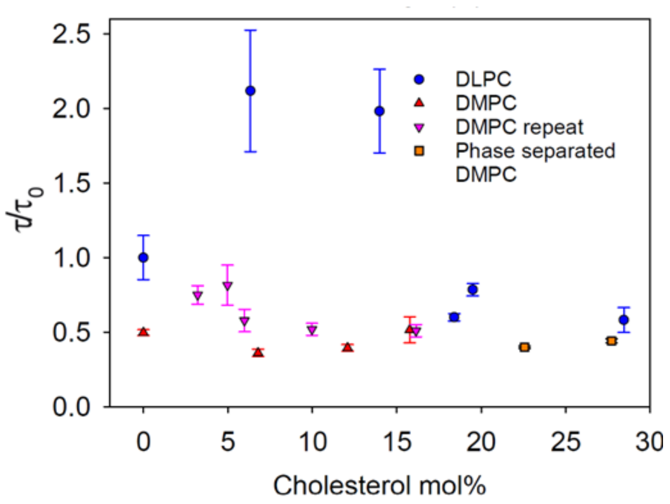

Figure S2. Effect of cholesterol content on GlpG-catalyzed LacYTM2 cleavage. The characteristic time constant τ that describes the cleavage reaction was calculated from the kinetic assay using FRET on the LacYTM2 model substrate and plotted against cholesterol concentration. The cholesterol concentration is measured from HPTLC lipid analysis. All τ values were normalized to the thinnest membrane DLPC with 0 mol% cholesterol (τ_0). All measurements were taken at 37°C.

a)

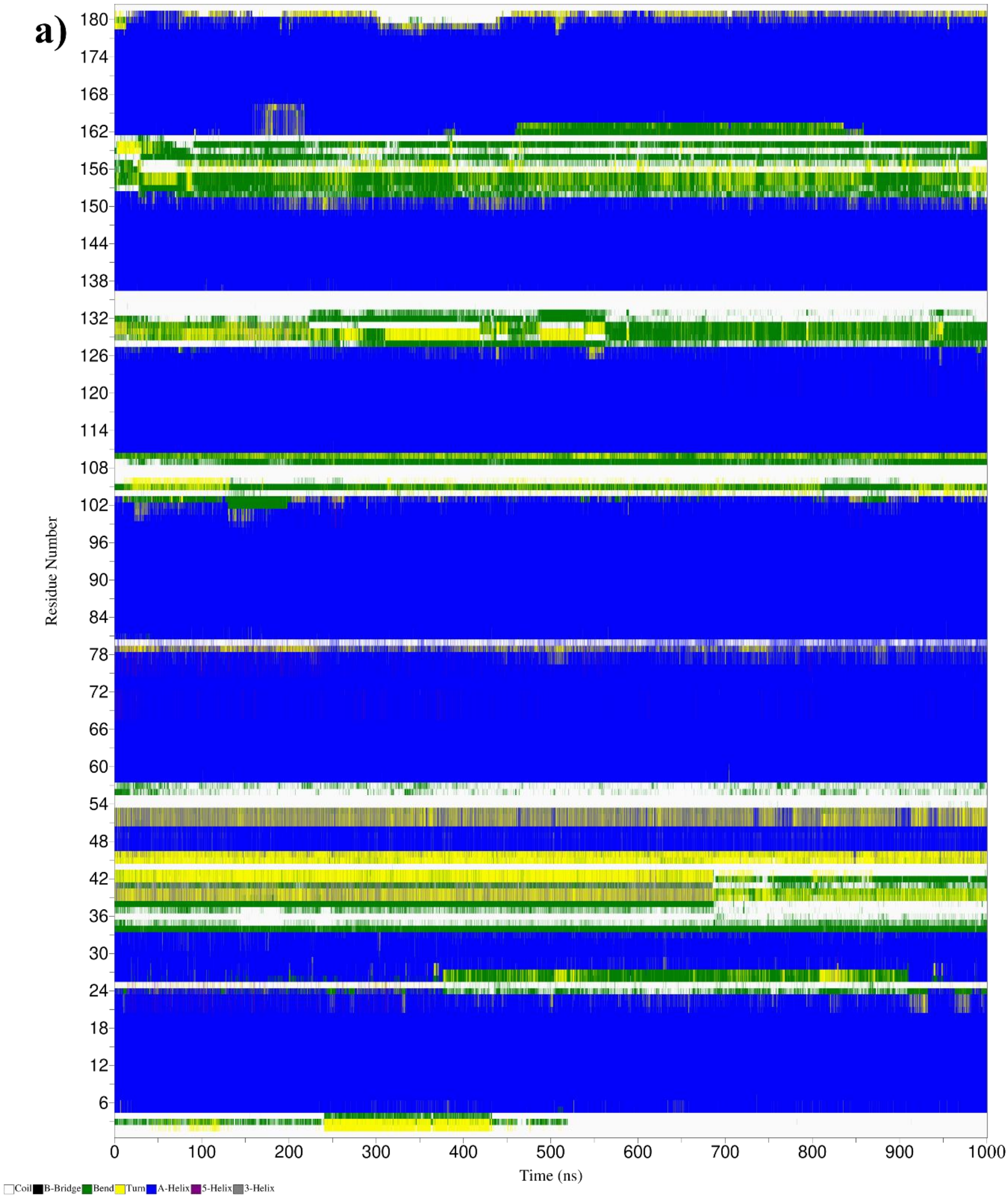
Residue Number
180
174
168
162
156
150
144
138
132
126
120
114
108
102
96
90
84
78
72
66
60
54
48
42
36
30
24
18
12
6
0
100
200
300
400
500
600
700
800
900
1000
Time (ns)
Coil
B-Bridge
Bend
Turn
A-Helix
5-Helix
3-Helix

b)

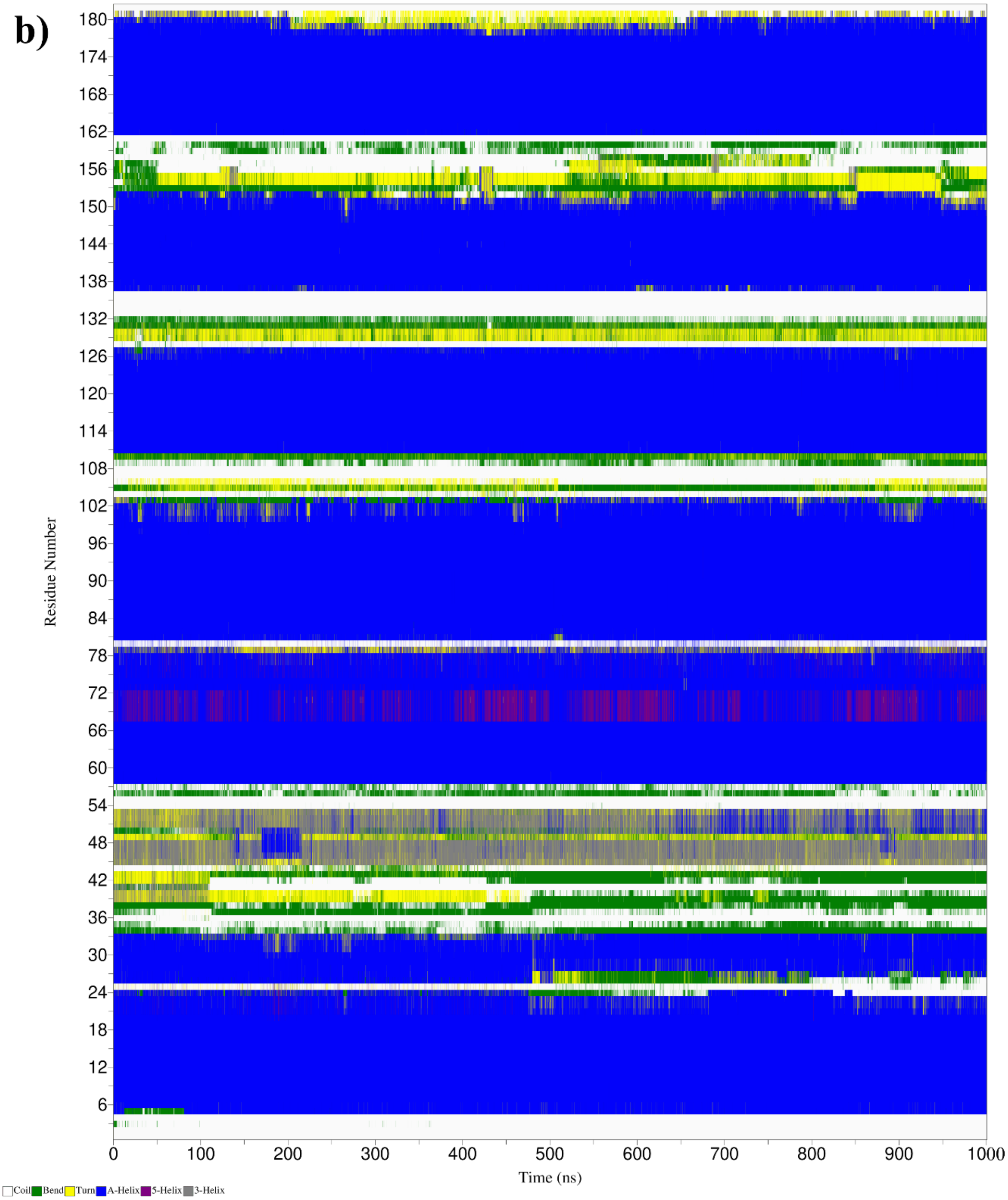

c)

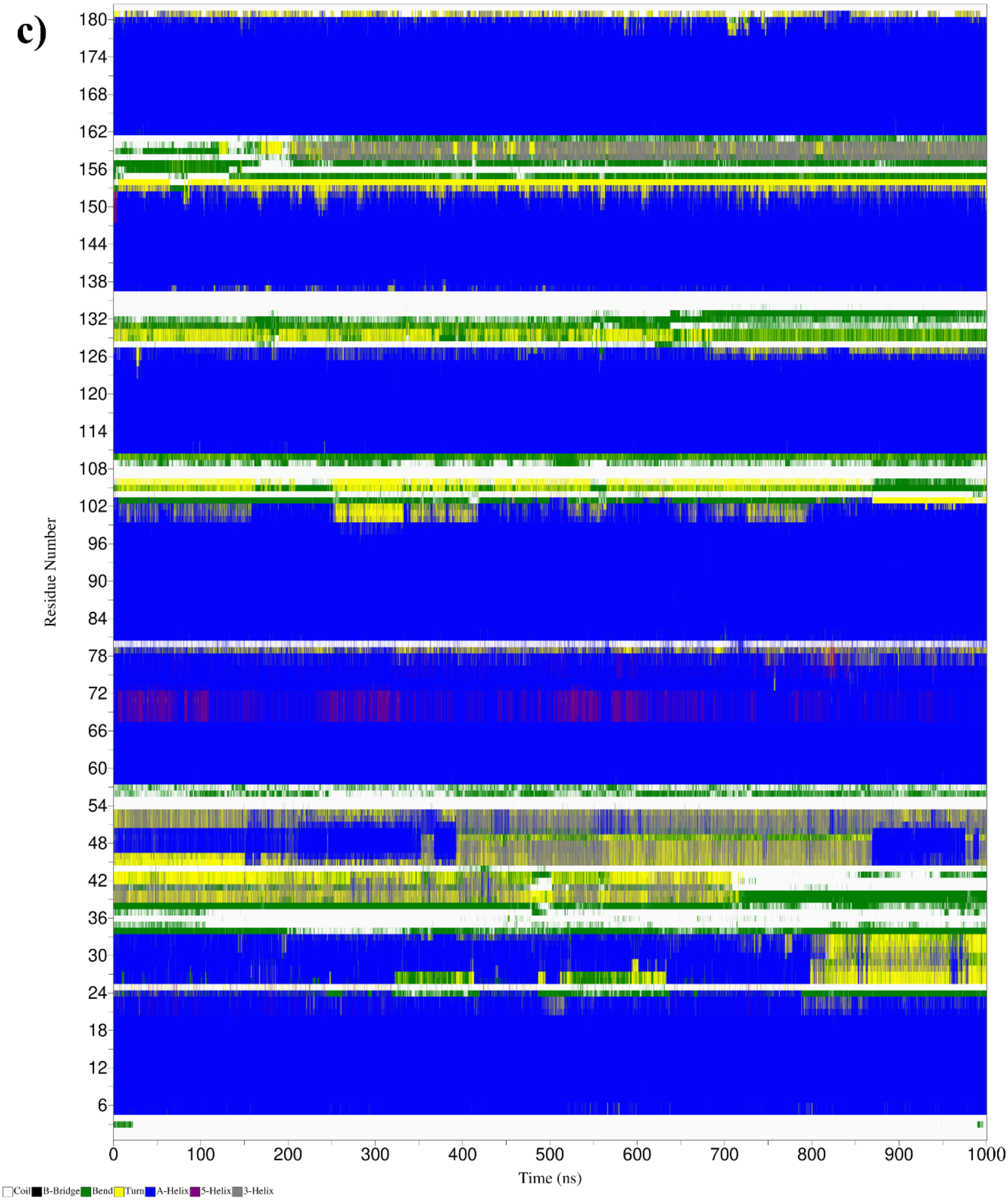

180
174
168
162
156
150
144
138
132
126
120
114
108
102
96
90
84
78
72
66
60
54
48
42
36
30
24
18
12
6
Residue Number
0
100
200
300
400
500
600
700
800
900
1000
Time (ns)
Coil
B-Bridge
Bend
Turn
A-Helix
5-Helix
3-Helix

d)

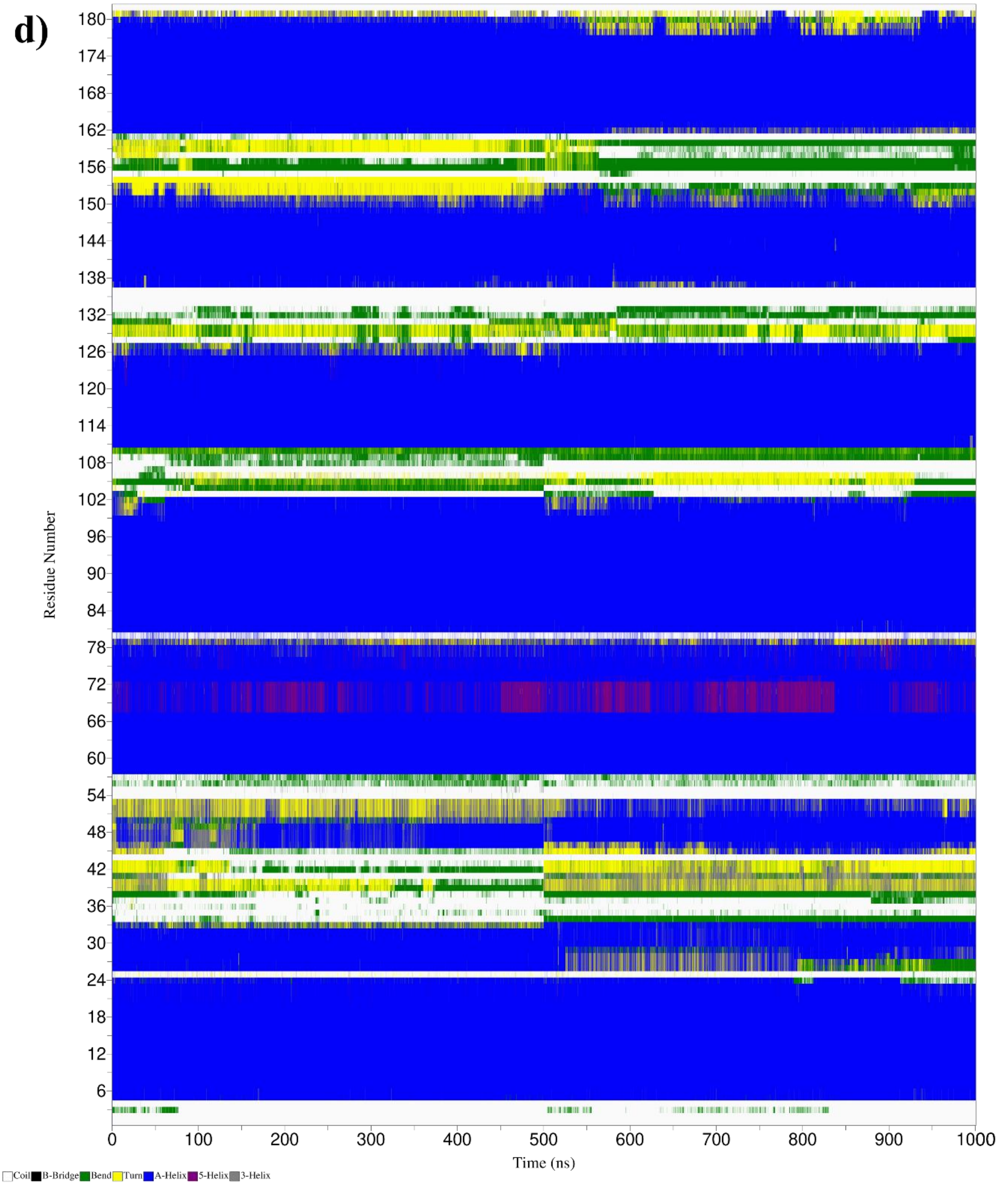
180
174
168
162
156
150
144
138
132
126
120
114
108
102
96
90
84
78
72
66
60
54
48
42
36
30
24
18
12
6
Residue Number
0
100
200
300
400
500
600
700
800
900
1000
Time (ns)
Coil
B-Bridge
Bend
Turn
A-Helix
5-Helix
3-Helix

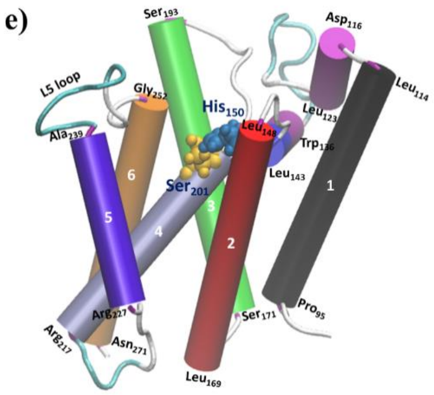

Figure S3. Secondary structure analysis of GlpG protein in DLPC systems at **a)** low cholesterol, **b)** high cholesterol, and in DMPC systems at **c)** low cholesterol and **d)** high cholesterol concentrations by dssp algorithm, **e**) Initial configuration of GlpG protein. GlpG contains six helices, which are labeled from 1 to 6, and contains 182 residues, starting from residue number 1 (Glu_{91}) to 182 (Ala_{272})*. The catalytic active center Ser_{201} (Residue no. 111) is on helix 4 (ice blue), and His_{150} (Residue no. 60) is on helix 2. Alterations for DLPC at low cholesterol are mainly found in regions 36-54, 68-72, and 153-161.

*Add 90 to each residue number in the secondary structure to match the literature residue number of respective amino acids in the sequence.

TM 1

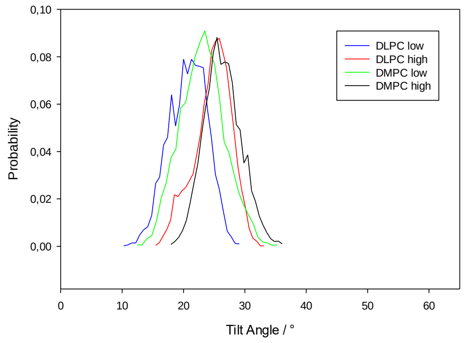

TM2

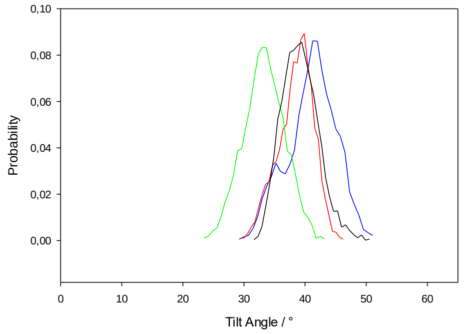

TM3

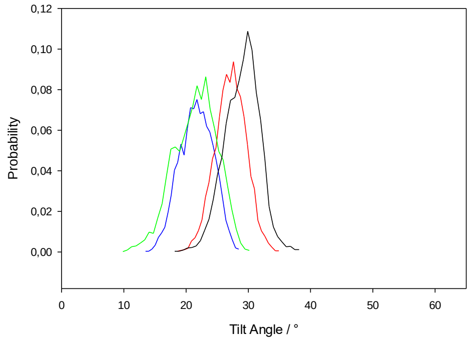

TM4

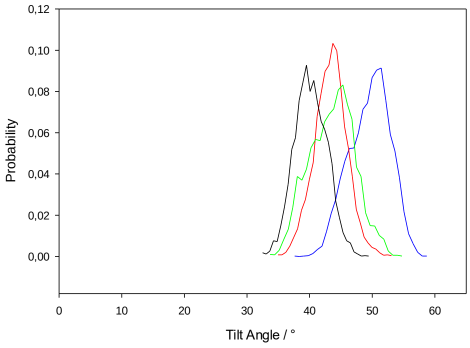

TM5

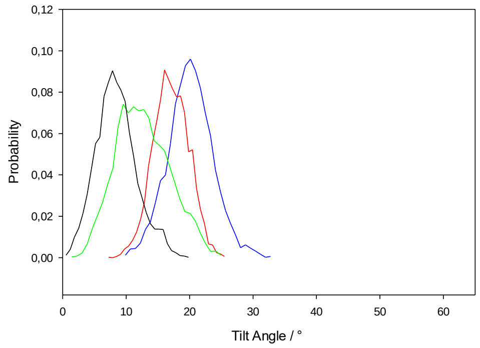

TM6

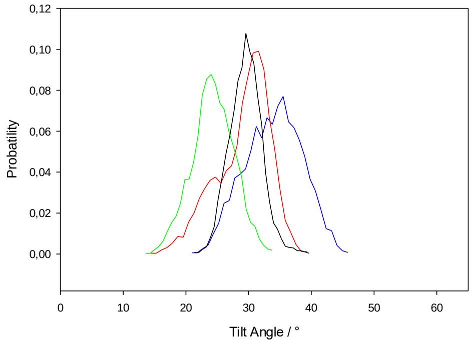

Figure S4. Histograms of the tilt angles of all transmembrane helices of GlpG in DLPC membranes at low (5%, blue curves) and high (30%, red curves) and in DMPC membranes at low (6%, green curves) and high (23%, black curves) cholesterol content calculated from the MD simulations.

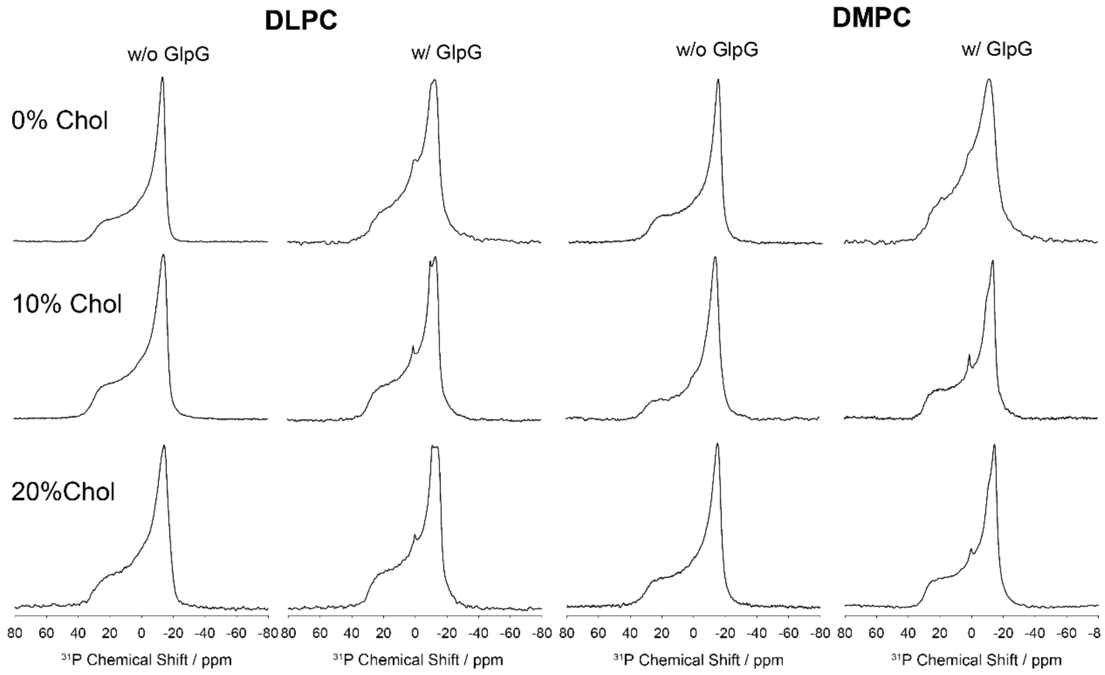

Figure S5. ^{31}P NMR spectra of all samples recorded from multilamellar vesicles in the absence and in the presence of GlpG at excess hydration and a temperature of 37°C at cholesterol concentrations as prepared.

DLPC at low cholesterol content (5%)

Annular cholesterol | Annular DLPC

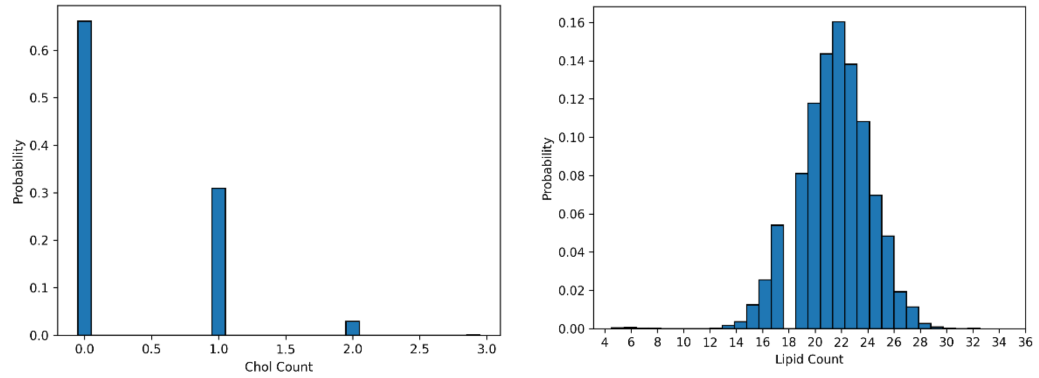

DLPC at high cholesterol content (30%)

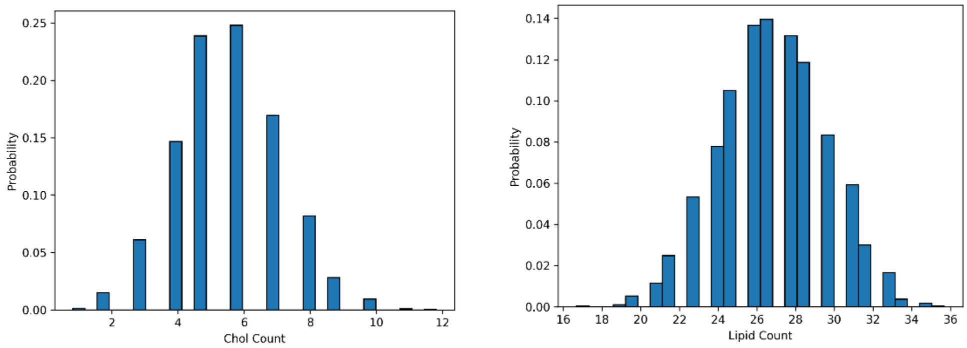

DMPC at low cholesterol content (6%)

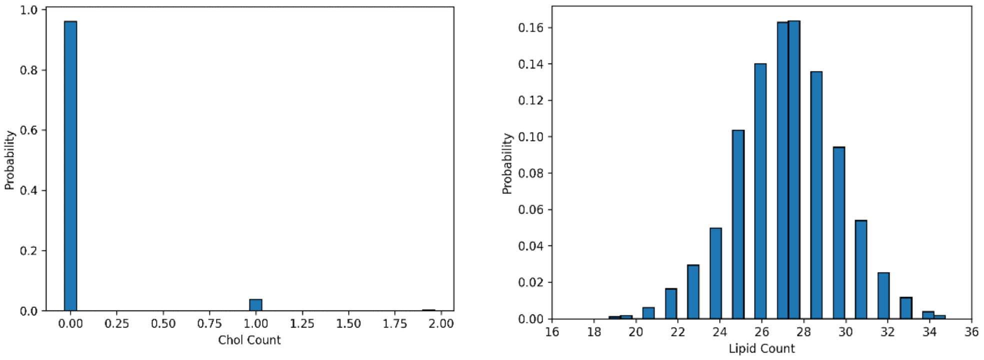

DMPC at high cholesterol content (23%)

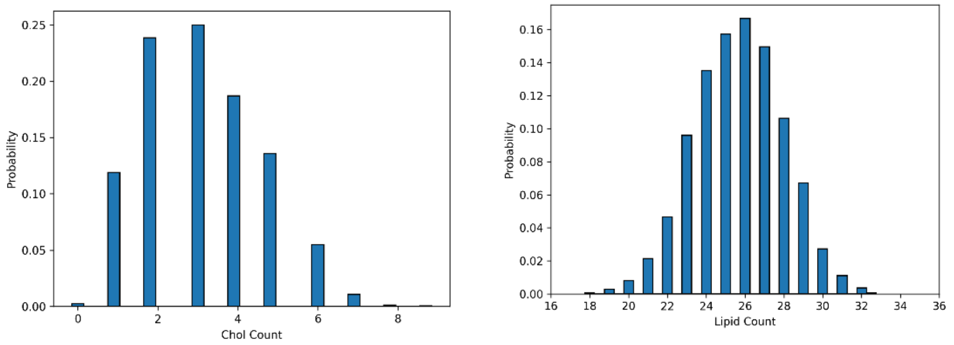

Figure S6. Histograms of the distribution of annular lipids (cholesterol vs. PC).

sn-2 chain order parameters for DLPC *sn*-2 chain order parameters for DMPC

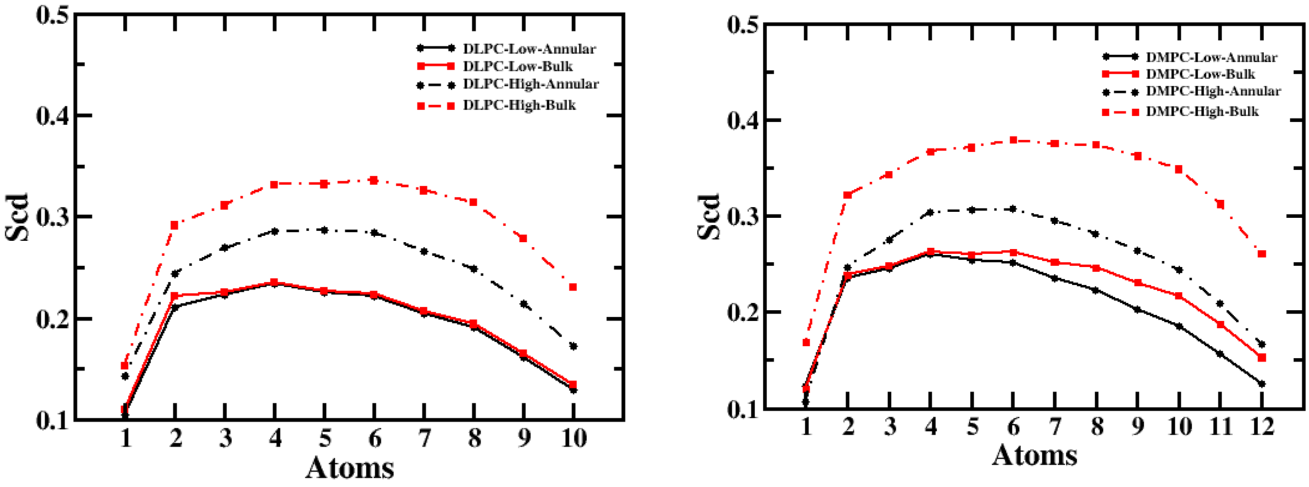

Figure S7. Lipid chain order parameters for the *sn*-2 chain of DLPC and DMPC at low and high cholesterol content in the presence of GlpG for the annular and bulk lipids calculated from the MD trajectories.

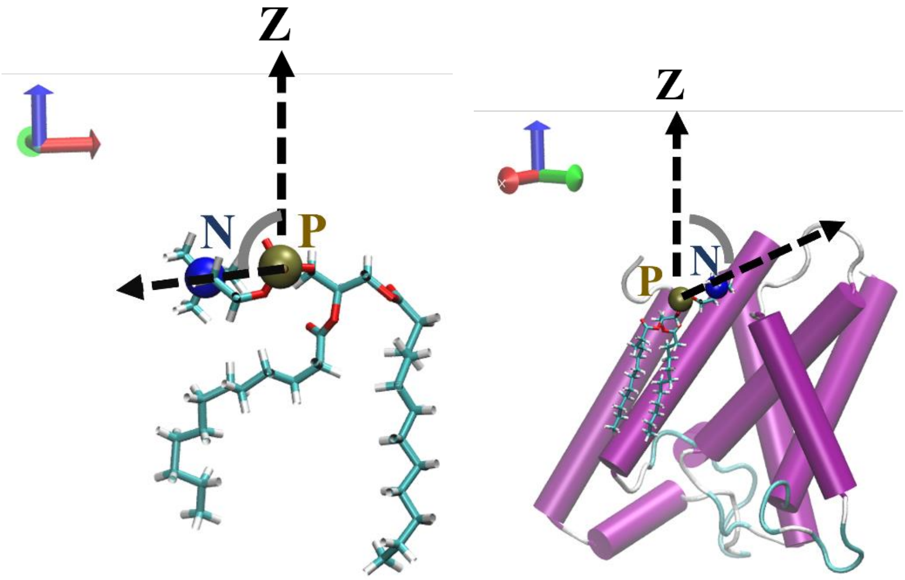

Figure S8. Pictorial representation of tilt angle computed between the lipid head group (P-N) vector and the bilayer normal for the bulk (left) and the annular lipids (right).

Table S1. Cholesterol concentration, Average order parameters (S_{CD}), projected chain lengths (*Lc*), number of *gauche* defects per chain, and area per molecule for deuterated DLPC and DMPC in the presence and absence of different GlpG at pH 4 at 37°C. Values for cholesterol-free membranes are reproduced from (17) with permission. NA = not applicable.

Phospholipid	Measured chol mol%	GlpG	S_{CD}	Lc (Å)	L/P ratio	SEM for L/P ratios	# gauche defects	A/Å2
**DLPC-*d*$_{46}$**	0.0	-	0.150	9.7			4.31	65.8
**DLPC-*d*$_{46}$/Chol**	7 ± 0.2	-	0.162	10.0			3.64	63.8
**DLPC-*d*$_{46}$/Chol**	13.4 ± 1.6	-	0.172	10.2			3.41	62.2
**DLPC-*d*$_{46}$/Chol**	19.4 ± 0.6	-	0.191	10.7			2.98	59.5
**DLPC-*d*$_{46}$/Chol**	22.5 ± 0.6	-	0.210	11.1			2.61	57.3
**DLPC-*d*$_{46}$/Chol**	28.6 + 1.3	-	0.235	11.6			2.15	54.8
**DLPC-*d*$_{46}$**	0.0	+	0.160	9.90	118	7	3.70	64.2
**DLPC-*d*$_{46}$/Chol**	8.8 ± 0.2	+	0.170	10.2	38.7	0.9	3.45	62.5
**DLPC-*d*$_{46}$/Chol**	15.3 ± 1.6	+	0.179	10.4	47.3	2.3	3.24	61.1
**DLPC-*d*$_{46}$/Chol**	23.71 + 0.5	+	0.192	10.7	44.3	0.6	2.98	59.5
**DLPC-*d*$_{46}$/Chol**	26.9 ± 2.6	+	0.201	10.9	54.8	1.8	2.81	58.5
**DLPC-*d*$_{46}$/Chol**	30.9 ± 1	+	0.210	11.1	48.9	1.1	2.64	57.5
**DMPC-*d*$_{54}$**	0	-	0.165	11.7			4.37	63.4
**DMPC-d$_{54}$/Chol**	6.4 ± 0.4	-	0.191	12.4			3.71	59.7
**DMPC-d$_{54}$/Chol**	10.9 ± 0.9	-	0.194	12.5			3.65	59.4
**DMPC-d$_{54}$/Chol**	12.9 ± 0.67	-	0.232	13.4			2.79	55.2
**DMPC-d$_{54}$/Chol**	18.9 ± 0.85	-	0.256	13.9			2.34	53.2
**DMPC-d$_{54}$/Chol**	26.9 + 1.2	-	NA	NA			NA	NA
**DMPC-*d*$_{54}$**	0.0	+	0.165	11.7	55	2	4.37	63.4
**DMPC-d$_{54}$/Chol**	7.3 ± 0.2	+	0.184	12.245	125.9	5.7	3.88	60.6
**DMPC-d$_{54}$/Chol**	8.9 ± 1.6	+	0.204	12.747	84.7	4.3	3.42	58.2
**DMPC-d$_{54}$/Chol**	14.1 ± 0.5	+	0.218	13.077	49.1	1.3	3.12	56.7
**DMPC-d$_{54}$/Chol**	15.6 ± 2.6	+	0.245	13.691	104.5	3.4	2.56	54.2
**DMPC-d$_{54}$/Chol**	23.4 ± 1	+	NA	NA	66.5	4.1	NA	NA

Table S2. The detailed simulation setup of systems in presence (*w GlpG*) and absence (*w/o GlpG*) of protein at low and high cholesterol (Chol) concentrations.

Systems	P/L ratio	% Chol	No. of Chol	No. of Phospholipids	No. of NaCl	No. of Water
**DLPC-Low** *(w/ GlpG)*	1:125	5	6	119	11	3823
**DLPC-Medium** *(w/ GlpG)*	1:125	16	20	105	10	3678
**DLPC-High** *(w/ GlpG)*	1:125	30	37	88	10	3518
**DMPC-Low** *(w/ GlpG)*	1:125	6	8	117	9	3436
**DMPC-Medium** *(w/ GlpG)*	1:125	15	19	106	9	3545
**DMPC-High** *(w/ GlpG)*	1:125	23	29	96	9	3460
**DLPC-Low** *(w/o GlpG)*	0:125	5	6	119	9	2375
**DLPC-High** *(w/o GlpG)*	0:125	30	36	89	5	2127
**DMPC-Low** *(w/o GlpG)*	0:125	6	8	117	5	2212
**DMPC-High** *(w/o GlpG)*	0:125	23	29	96	4	2108

Table S3. Analysis of bulk vs. annular lipids in the different system based on the histograms shown in Fig. S6 from the MD simulations.

System	Bulk lipids		Annular lipids	
	Cholesterol/phospholipid	Cholesterol content	Cholesterol/phospholipid	Cholesterol content
DLPC low	6/97	5.8%	0/22	0%
DLPC intermediate	19/78	19.6%	1/27	3.6%
DLPC high	31/61	33.7%	6/27	18.2%
DMPC low	8/90	8.2 %	0/27	0%
DMPC intermediate	17/80	17.5%	2/26	7.1%
DMPC high	26/70	27.1%	3/26	10.3%

Table S4: Cholesterol concentration, normalized τ-values, lipid/protein ratio (L/P) and substrate/protein ratio (S/P) of DLPC and DMPC in the presence of GlpG. NA = not applicable.

Phospholipid	Measured chol mol%	Normalized τ	SEM for normalized τ	Lc (Å)	L/P ratio	SEM for L/P ratio	S/P ratio
**DLPC**	0.0	1	0.15	9.9	82.7	5.7	0.55
**DLPC**	6.3 ± 0.4	2.12	0.41	10.1	92.3	4.7	0.56
**DLPC**	14.0 ± 0.5	1.98	0.28	10.4	109.4	6.2	0.62
**DLPC**	18.4 ± 2.1	0.60	0.02	10.5	69.5	4.5	0.62
**DLPC**	19.5 ± 0.3	0.79	0.04	10.6	74.9	4.6	0.61
**DLPC**	28.4 ± 1.9	0.58	0.08	10.9	74.7	3.8	0.69
**DMPC**	0.0	0.50	0.02	11.6	123.7	5.1	0.69
**DMPC**	6.8 ± 0.2	0.36	0.02	12.4	103.9	23.1	0.44
**DMPC**	12.1 ± 1.2	0.40	0.02	13.0	86.5	10.0	0.41
**DMPC**	15.8 ± 1.0	0.52	0.09	13.5	128.4	25.5	0.52
**DMPC**	22.6 ± 1.4	0.40	0.01	NA	98.9	5.5	0.60
**DMPC**	27.7 ± 1.7	0.44	0.01	NA	83.5	8.7	0.65
**DMPC repeat**	3.2 ± 0.1	0.82	0.13	12.0	49.4	6.3	1.18
**DMPC repeat**	5 ± 0.1	0.58	0.08	12.2	49.4	6.8	1.17
**DMPC repeat**	5.0 ± 0.4	0.52	0.04	12.3	56.7	3.0	1.19
**DMPC repeat**	10.0 ± 0.8	0.51	0.04	12.8	43.1	8.3	1.20
**DMPC repeat**	16.1 ± 0.5	0.69	0.06	13.5	38.4	12.2	0.92
