# Supplementary material for: Evaluating the impact of the membrane thickness on the function of the intramembrane protease GlpG
Source: Biophys J. 2024 Nov 1;123(23):4067–81. doi: 10.1016/j.bpj.2024.10.019 (PMC11628809; doi:10.1016/j.bpj.2024.10.019)
Supplement: Document S2. Article plus supporting material [file mmc2.pdf]

Biophysical Journal
Article

Biophysical Society

Evaluating the impact of the membrane thickness on the function of the intramembrane protease GlpG

Oskar Engberg,[1] Anjana V. Mathath,[2] Viola Döbel,[1] Christian Frie,[3] Marius K. Lemberg,[3] Debashree Chakraborty,[2] and Daniel Huster[1,*]
[1]Institute for Medical Physics and Biophysics, University of Leipzig, Leipzig, Germany; [2]Biophysical and Computational Chemistry Laboratory, Department of Chemistry, National Institute of Technology Karnataka, Mangalore, Karnataka, India; and [3]Center for Biochemistry and Cologne Excellence Cluster on Cellular Stress Responses in Aging-Associated Diseases (CECAD), University of Cologne, Cologne, Germany

ABSTRACT Cellular membranes exhibit a huge diversity of lipids and membrane proteins that differ in their properties and chemical structure. Cells organize these molecules into distinct membrane compartments characterized by specific lipid profiles and hydrophobic thicknesses of the respective domains. If a hydrophobic mismatch occurs between a membrane protein and the surrounding lipids, there can be functional consequences such as reduced protein activity. This phenomenon has been extensively studied for single-pass transmembrane proteins, rhodopsin, and small polypeptides such as gramicidin. Here, we investigate the *E. coli* rhomboid intramembrane protease GlpG as a model to systematically explore the impact of membrane thickness on GlpG activity. We used fully saturated 1,2-dilauroyl-sn-glycero-3-phosphocholine (DLPC) and 1,2-dimyristoyl-sn-glycero-3-phosphocholine(DMPC) model lipids and altered membrane thickness by varying the cholesterol content. Physical membrane parameters were determined by ^{2}H and ^{31}P NMR spectroscopy and correlated with GlpG activity measurements in the respective host membranes. Differences in bulk and annular lipids as well as alterations in protein structure in the respective host membranes were determined using molecular dynamics simulations. Our findings indicate that GlpG can influence the membrane thickness in DLPC/cholesterol membranes but not in DMPC/cholesterol membranes. Moreover, we observe that GlpG protease activity is reduced in DLPC membranes at low cholesterol content, which was not observed for DMPC. While a change in GlpG activity can already be due to smallest differences in the lipid environment, potentially enabling allosteric regulation of intramembrane proteolysis, there is no overall correlation to cholesterol-mediated lipid bilayer organization and phase behavior. Additional factors such as the influence of cholesterol on membrane bending rigidity and curvature energy need to be considered. In conclusion, the functionality of α-helical membrane proteins such as GlpG relies not only on hydrophobic matching but also on other membrane properties, specific lipid interaction, and the composition of the annular layer.

SIGNIFICANCE The function of membrane proteins depends on lipid membrane properties. An obvious condition is the hydrophobic matching between host membrane and embedded membrane proteins. Here, we systematically studied the effect of membrane thickness of model membranes composed of phosphatidylcholines with acyl chains of 12 or 14 carbons with a cholesterol gradient on the function of the *E. coli* protease GlpG. GlpG changes its substrate cleavage velocity in response to the hydrophobic thickness of the membrane, but specific cholesterol concentrations in DLPC membranes inhibit GlpG function. It is important to understand how the combination of cholesterol, membrane thickness, bending rigidity, and curvature energy can modulate membrane protein function, as demonstrated in this study.

INTRODUCTION

The lipid composition of mammalian plasma membranes is very heterogeneous with over a thousand different lipid species (1). This diversity is responsible for variations in many membrane properties such as curvature, fluidity, phase behavior, domain structure, and hydrophobic thickness (2,3). Membrane proteins are embedded in this lipid bilayer and their function can be affected by individual lipid species such as cholesterol, phosphatidylinositol-4,5-bisphosphate, or phosphatidylserine (4–7), but also by the bulk properties of the surrounding lipid environment (8,9). A well-accepted concept to describe membrane-protein interaction is hydrophobic matching (10–12). The most efficient incorporation of proteins is encountered when lipid bilayers and membrane proteins have similar hydrophobic thicknesses to

Submitted July 12, 2024, and accepted for publication October 30, 2024.
*Correspondence: daniel.huster@medizin.uni-leipzig.de
Editor: Leonid Brown.
https://doi.org/10.1016/j.bpj.2024.10.019
© 2024 The Author(s). Published by Elsevier Inc. on behalf of Biophysical Society.
This is an open access article under the CC BY license (http://creativecommons.org/licenses/by/4.0/).

avoid unfavorable energetic contributions due to exposure of hydrophobic segments to the aqueous environment. If the hydrophobic thickness of the membrane does not match the dimension of transmembrane proteins, either the protein or the membrane or both have to adapt (13). For instance, the lipids of the annular layer can adjust by increasing or decreasing their acyl chain order to either increase or decrease the bilayer thickness. Alternatively, membrane proteins can tilt, aggregate, or dimerize/oligomerize to minimize the hydrophobic mismatch (14). In addition, membrane proteins can adjust their α-helical content as observed for bovine rhodopsin reconstituted in lipid bilayers of varying thickness (15). It is assumed that these phenomena would modulate protein activity. Also, physical properties of the membrane determine sorting and organization of membrane protein. While of fundamental importance, only few examples have described the impact of the membrane composition on membrane protein localization and function (16–22).

We could recently show that the activity of the rhomboid protease GlpG is modulated by the surrounding membrane environment, in particular the hydrophobic thickness of the lipid bilayer (17), but also show some headgroup specificity (23). Intramembrane proteases cleave helical transmembrane proteins, and the proteolysis occurs in the plane of the membrane (24). They are interesting systems to investigate the influence of the membrane on protein function. Not only is the enzyme activity dependent on the properties of the membrane environment, it has also been suggested that rhomboid family proteins actively thin lipid membranes to tune their activity. Thereby, they may overcome the viscosity-imposed diffusion limit that represents a rate-limiting step in the enzyme interacting with its substrate (25). In a related manner, rhomboid proteases and catalytically inactive pseudoprotease homologs have been suggested to introduce a thinned membrane region tailored for their interaction to protein clients and substrates (26). Although membrane thinning was not directly observed in these studies, molecular dynamics (MD) simulations showed that the GlpG rhomboid protease actively thins lipid membranes more or less irrespective of their lipid composition (27,28). The phenomenon of lipid thinning was explained by the relatively small hydrophobic thickness of GlpG revealed in the crystal structure (29) in comparison with the thickness of the host membrane. So instead of the protein adapting to the properties of the surrounding lipid bilayer, the membrane thickness is modulated by the protein. We could show this lipid-modulating capacity of GlpG experimentally using ^{2}H NMR spectroscopy precisely measuring membrane thickness (17). However, we also observed an intriguing headgroup specificity of the effect: while palmitoyl-oleoyl-phosphatidylcholine (POPC) membranes are not thinned by GlpG, the thickness of phosphatidylethanolamine/phosphatidylglycerol (POPE/POPG) bilayers was reduced by 8%. This result was at first sight surprising considering that POPC and POPE/POPG membranes display the same glycerol/acyl chain structure. But, of course, PE lipids with their negative intrinsic curvature are known to cause an increased hydrophobic thickness of PE-containing membranes (30) and have also been identified as important bulk molecules for protein function (31,32). In accordance with these findings, mixtures of POPC/POPG would not show the membrane thinning effect of GlpG (17).

When analyzing the activity of GlpG in various membranes, another interesting result was observed (17): GlpG showed fastest substrate cleavage in completely artificial 1,2-dimyristoyl-*sn*-glycero-3-phosphocholine(DMPC) membranes. Enzyme reaction was slightly slower in POPC bilayers, which is a physiologically very relevant lipid, but not found in *E. coli* membranes. In an *E. coli*-relevant lipid mix (POPE/POPG), GlpG catalysis was again slower than in POPC. Adding the *E. coli* lipids POPE/POPG to GlpG reconstituted in DMPC even showed an inhibitory effect. Enzyme reaction was also much slower in 1,2-dilauroyl-sn-glycero-3-phosphocholine (DLPC) and almost completely lost in dipalmitoylphosphatidylcholine/cholesterol bilayers. This underlines the sensitivity of GlpG activity to the (thickness of the) surrounding membrane, which was also found in another recent study (23). We suggested that there is an optimum hydrophobic thickness for the GlpG catalytic cycle that corresponds to ∼24–26 Å. Since free-standing POPE/POPG membranes show a higher hydrophobic thickness due to the negative intrinsic curvature, GlpG actively thins these bilayers most likely due to specific interactions with the PE headgroups as concluded from ^{31}P NMR spectra (17), also indicating the headgroup specificity in protein-lipid interactions.

These interesting results leave important open questions. In the aforementioned experimental and MD studies, quite different membrane systems were compared, using molecules varying in their headgroup structure, chain composition, and degrees of chain unsaturation. Therefore, here we report a systematic study on the activity of GlpG as a function of hydrophobic thickness of the membrane applying a gradual variation in bilayer thickness. There are several mechanisms that can regulate membrane thickness, e.g., temperature (33), variation in phospholipid headgroup or chain length (34–36), the inclusion of small molecules (37), detergents (38), and cholesterol (39). Temperature is an obvious parameter that can affect membrane thickness (33), but would also change the kinetics of the enzyme reaction independent of bilayer thickness. Changes in phospholipid headgroup, e.g., from a larger PC to a smaller PE headgroup, would result in different membrane thicknesses. However, it could also affect enzyme activity independently because of PE's spontaneous curvature and hydrogen bonding capacity, as observed, e.g., for rhodopsin (40,41). Small molecules and detergents can thin the membrane, but a concentration gradient would not necessarily lead to a linear variation of bilayer thickness and

high concentration could reduce liposome structural integrity (42). Cholesterol is well known to increase membrane thickness through its condensation effect and its concentration can easily be fine-tuned leading to a well-controlled increase in membrane thickness (43). However, cholesterol not only affects bilayer thickness and area per lipid, it also influences the energetics of membrane elastic deformation and curvature (44,45). Both could also be important properties with regard to the modulation of protein function by the surrounding lipid environment.

While our membrane models are based on a modular approach that considers important parameters such as membrane thickness, intrinsic curvature, elasticity, etc., often as isolated quantities, the correlation between all these model parameters is neglected in first approximation. When designing the experiments for the current study, we followed this “divide and conquer” approach. To keep lipid properties as comparable as possible, we investigated GlpG activity in mixtures of cholesterol with saturated PC molecules. Although not found naturally in *E. coli*, cholesterol is a very useful molecule to systematically vary bilayer thickness. By using different cholesterol concentrations, the effect of hydrophobic thickness on GlpG function, both its effect on the bilayer thickness and the model substrate cleavage kinetics of GlpG, was evaluated. We tested both the impact of GlpG on membrane thickness and other membrane properties as well as model substrate cleavage in disaturated PC membranes of 12 or 14 carbons in the acyl chains (DLPC and DMPC). The combination of experimental ^{2}H NMR and MD simulations provides unique insights into the interesting adaptation process of the lipid membrane to GlpG.

MATERIALS AND METHODS

The lipids DLPC, 1,2-dilauroyl-d_{46}-*sn*-glycero-3-phosphocholine (DLPC-d_{46}), DMPC, 1,2-dimyristoyl-d_{54}-*sn*-glycero-3-phosphocholine (DMPC-d_{54}), and 1,2-diheptanoyl-*sn*-glycero-3-phosphocholine (DHPC), as well as cholesterol, were purchased from Avanti Polar Lipids (Alabaster, AL). The LacYTM2 substrate with the sequence KRHDINE(EDANS)ISK SDTGK(DABCYL)IFAAI SLFSLLFQPL FGLLSKK was synthesized in the Core Unit Peptide Technologies at the University of Leipzig. Organic solvents used for lipid extraction and quantification were HPLC grade. A lipid extruding kit was bought from Lipex Biomembranes (Vancouver, BC, Canada). *n*-Dodecyl-β-D-maltoside (DDM) was purchased from Glycon (Luckenwalde, Germany). Milli-Q water deionized to 18.2 mΩ cm^{-1} was used for all experiments and was obtained from a Milli-Q gradient system (Merck Millipore, Darmstadt, Germany). All other chemicals were bought from Merck-Sigma (Taufkirchen, Germany).

Recombinant expression of GlpG

The 25b(+)-based (Novagen) expression vector with hexahistidine-tagged *E. coli* GlpG has been described previously (17). Chemically competent BL21(DE3)pLysS cells were transformed with the expression vector, grown in LB medium containing 100 μg/mL ampicillin and 34 μg/mL chloramphenicol at 37°C, and expression of GlpG was induced by adding 0.3 mM IPTG at OD_{600} of 0.3 followed by overnight (~20 h) expression at 16°C. Cells were harvested by centrifugation at 6000 × *g* for 30 min at 4°C and resuspended in 20 mM HEPES/KOH (pH 7.4), 5 mM $MgCl_2$, 150 mM NaCl, 10% glycerol, 1 mM phenylmethylsulfonyl fluoride (PMSF), 5 mM β-mercaptoethanol. Before performing the lysis, 200 μg/mL lysozyme, 1 mM PMSF, and benzonase (37.5 units of 2.5 ku for 1 L *E. coli* culture, Merck KGaA, Darmstadt, Germany) were added and cells were lysed using Emulsiflex (Avestin, Ottawa, ON, Canada) two cycles with a maximum pressure of 15 kpsi (100 MPa). The lysate was centrifuged at 100,000 × *g* for 45 min at 4°C and the obtained membrane pellet was resuspended in 10 mL 50 mM HEPES/KOH (pH 7.4), 5 mM $MgCl_2$, 150 mM NaCl, 10% glycerol, 1 mM PMSF, 5 mM β-mercaptoethanol using a Dounce-homogenisator on ice. GlpG-His_6 was solubilized by 1.5% DDM on a rotating wheel for 1 h at 4°C and subsequently separated from membrane debris by centrifugation at 86,000 × *g* for 1 h at 4°C. Cleared protein extract was added to Ni-NTA beads (Macherey-Nagel, Düren, Germany) and batch incubated for 1 h on a rotating wheel at 4°C for His-tag affinity purification. Bound GlpG-His_6 was first washed with 5 column volumes (cv) 50 mM HEPES/KOH, 300 mM NaCl, 10% glycerol, 0.05% DDM (pH 8). A further wash was done with 5 cv 50 mM HEPES/KOH, 300 mM NaCl, 10% glycerol, 0.05% DDM, 50 mM imidazole (pH 8), and a final wash with 5 cv 50 mM HEPES/KOH, 300 mM NaCl, 10% glycerol, 0.05% DDM (pH 8). The elution was performed by adding 15 mL 50 mM acetate, 300 mM NaCl, 10% glycerol, 0.05% DDM (pH 4.5). Eluates were collected in 1 mL aliquots. Purification of recombinant GlpG was analyzed by SDS-PAGE using 12% Tris-glycine polyacrylamide gels, which were visualized through Coomassie staining using an ImagQuant 800-System (Cytiva).

Reconstitution of GlpG into lipid membranes

Phospholipids and cholesterol were combined at the respective mixing ratios in a 1:1 (v/v) ratio of chloroform/MeOH and evaporated using a rotary evaporator at 40°C until dry. The resulting dried samples were dispersed in buffer (50 mM sodium acetate, 150 mM NaCl [pH 4]) at a concentration of 10 mg/mL lipid. To produce large unilamellar vesicles, the lipid dispersions were extruded through 2 stacked 100 nm polycarbonate filters using the lipid extruder (Lipex Biomembranes) as described in the literature (46).

For the formation of bicelles, DHPC was added in a 6-fold molar excess to the liposome dispersion. After incubation at 50°C for 30 min, a clear solution was obtained. For substrate cleavage assay, the peptide substrate LacYTM2 was dissolved to 1 mg/mL in buffer (50 mM sodium acetate, 150 mM NaCl, 10 mg/mL DHPC, 0.05 wt % DDM [pH 4]) and added to the bicelle solution. GlpG (in DDM) was added to the bicelles maintaining a substrate/GlpG molar ratio of 1.2 in 50 mM acetate, 300 mM NaCl, 10% (v/v) glycerol, 0.05% DDM (pH 4.5). If required, samples were diluted to a concentration of 0.5–1 mg/mL GlpG using buffer at pH 4 including an extra 0.05 wt % DDM to enhance protein stability. Samples without GlpG were diluted to achieve the same volume using the detergent buffers.

The samples were then subjected to three cycles of incubation: 20 min in a 42°C water bath followed by 20 min on ice, to integrate GlpG and the substrate into the bicelles. Subsequently, 100 mg/mL of BioBeadsSM2 (Bio-Rad, Hercules, CA) was added, and the samples were shaken overnight at 4°C to form multilamellar vesicles (MLVs). This procedure was repeated for 4 h until the solution became turbid, typically requiring two biobead cycles. A 100 μm EASYstrainer filter (Greiner, Frickenhausen, Germany) was used to remove the biobeads, followed by centrifugation at 4000 × *g* for 20 min at 4°C. The resulting pellet which contained MLVs with reconstituted GlpG was resuspended in 1 ml pH 4 buffer. This lipid dispersion was centrifuged at 21,500 × *g* for 20 min at 4°C. The resulting pellets with the deuterated lipids were transferred into 4 mm NMR MAS rotors and stored at −20°C, and the pllets with the nondeuterated lipids was stored in Eppendorf containers at −20°C until usage in functional assays.

Solid-state NMR measurements

The ^{2}H NMR spectra were recorded on a Bruker 750 Avance I NMR spectrometer at a resonance frequency of 115.1 MHz. A 4 mm double channel

magic angle spinning probe was used, but no spinning was applied. For signal acquisition, a quadrupolar echo sequence with two $\pi/2$ pulses of 2.5–4 μs length separated by a 30 μs delay was used (47). The recycle delay was 1 s and the spectral width was $\pm$250 kHz. A Mathcad program was used to dePake the NMR spectra and obtain the order parameters and to export the spectra as described previously (30,48). The mean-torque model was used to calculate the project chain lengths (49).

Following ^{2}H NMR measurements, samples were subjected to ^{31}P NMR analysis using a Bruker Avance III 600 MHz spectrometer equipped with a 5 mm probe head with a designated ^{31}P channel (resonance frequency of 242.9 MHz) and ^{1}H decoupling (Bruker BioSpin, Ettlingen, Germany). A Hahn echo pulse sequence with a 10 μs 90° pulse length was employed for acquiring the ^{31}P NMR spectra. The acquisition parameters included an echo delay of 50 μs, a spectral width of 50 kHz, and a relaxation delay of 3 s. Low-power broadband ^{1}H decoupling was implemented during acquisition ($\omega_H/2\pi = 2.5$ kHz). All NMR measurements were conducted at 37°C.

A program written in Mathcad 14.0 (Parametric Technology Corporation, Needham, MA) was utilized to analyze the ^{31}P NMR spectra. This program calculates the ^{31}P NMR lineshape, enabling the estimation of the contributions for each anisotropic powder pattern, the extraction of the chemical shift anisotropy (CSA) ($\Delta\sigma$), and the shape of the vesicles. Lipids show a slight tendency to orient with the long axis perpendicular to the external magnetic field. In some lipid preparations, the vesicles were slightly deformed (50).

Protein and peptide quantification

The concentration of GlpG was determined by dissolving the MLV preparations in a detergent buffer (50 mM Tris, 150 mM NaCl, and 50 mM SDS [pH 7]) and measuring the absorption at 280 nm with a NanoDrop 1000 spectrometer (NanoDrop Technologies, Wilmington, DE). The Lambert-Beer law was used for calculating the protein concentration. The LacYTM2 peptide concentration was determined with the Pierce BCA Protein Assay Kit (Thermo Fisher Scientific, Waltham, MA) according to the manufacturer's protocol and measured on an Infinite M200 plate reader (Tecan, Männedorf, Switzerland). The concentration of the LacYTM2 (EDANS/DABCYL) kinetic peptide variant was determined by measuring the absorbance of the DABCYL group at 453 nm. The ultimate peptide concentration was derived from a calibration curve (linear model) using the LacYTM2 (EDANS/DABCYL) peptide dissolved in dimethyl sulfoxide.

Lipid quantification with HPTLC

Samples were resolved in a pH 7 buffer (50 mM Tris, 150 mM NaCl) or a pH 4 buffer (50 mM sodium acetate, 150 mM NaCl). Lipid extraction followed the Bligh and Dyer method. In short, 100 μL of a buffer-resolved MLV sample was mixed with 200 μL of $CHCl_3$/MeOH (1:1 [v/v]) for 30 s. Phase separation was achieved by centrifugation at 20°C and 10,000 $\times$ g for 5 min. The $CHCl_3$ phase containing the lipids was transferred to a vacuum centrifuge to remove the organic solvent by evaporation. For lipid quantification, the lipid film was dissolved in $CHCl_3$ and applied to a high-performance thin-layer chromatography (HPTLC) silica gel 60 plate (Merck KGaA) using a CAMAG Linomat 5 sample applicator (CAMAG, Berlin, Germany), along with a lipid calibration standard on the same HPTLC plate. TLC development took place in a glass chamber (CAMAG) with a mobile phase of chloroform/ethanol/water/triethylamine (30:35:7:35 [v/v/v/v]) for 2–3 h for PC lipids or 45 min for PE/PG lipids. Lipid spots were visualized by primuline staining in 200 mL of acetone/H_2O (4:1 [v/v]) followed by UV light excitation. Cholesterol quantification was done by changing the mobile phase to hexane/diethylether/acetic acid (80:20:1 [v/v/v]). Visualization of the cholesterol was done by using copper sulfate (10% [w/v]) in phosphoric acid (8% [v/v]) as staining solution. The plates were heated for 5–30 min until the lipid spots appeared black. The lipid concentration in the MLV samples was determined using the calibration curve from the lipid standard.

Enzymatic cleavage assays in membranes

For functional studies in membranes, both GlpG and the peptide substrate LacYTM2 (EDANS/DABCYL) were reconstituted at a molar ratio of 1:1.2 at pH 4, a condition where GlpG is inactive. Multilamellar lipid/protein vesicles were used for the measurements as described above. In the fluorescence assay, a GlpG concentration of 6 μM in a final volume of 50 μL at pH 7 was used at a temperature of 37°C. The progress of substrate cleavage was tracked over time by measuring EDANS fluorescence with a Spark plate reader (Tecan). The resulting progress curves were fitted to a single exponential model, $I(t) = I_{max} - I_0 \times \exp(-t/\tau)$, where $I(t)$ represents the EDANS fluorescence signal over time, I_0 is the initial fluorescence (offset), I_{max} is the maximum fluorescence, and τ is the characteristic time constant.

MD simulation

The coordinates of the 3D crystal structure of GlpG rhomboid protease was taken from the Protein Data Bank (chain A from PDB: 2NRF, resolution: 2.60 Å) (51). The transmembrane domain of GlpG consists of 182 residues (residue number starting from 91 to 272) and has 6 helical segments. The web server CHARMM-GUI (52) was used to embed and place the protein in various hydrated lipid environments. We simulated DLPC membranes at 5 mol % (low), 16 mol % (intermediate), and 30 mol % (high) cholesterol and DMPC membranes at 6 mol % (low), 15 mol % (intermediate), and 23 mol % (high) cholesterol concentrations in the presence and absence of GlpG protein. The total molar protein/lipid ratio was maintained at 1:125. The physiological salt concentration of 150 mM NaCl was added to each system. The details of the composition of the simulated lipid/protein systems are given in Table S2.

The MD simulations were carried out using GROMACS 2022.2 software package (53). The lipid molecules were parameterized by CHARMM-36 (54) with CMAP lipid parameters. The TIP3P water model (55) was used for solvating the system. Energy minimization was done for all the systems by the steepest-descent algorithm to remove the steric clash between lipid-protein and lipid-water molecules (53). The electrostatic interactions were estimated using the particle mesh Ewald algorithm (56) and the van der Waals interactions were calculated using a cutoff value of 1.2 nm. To restrain the bond associated with hydrogen atoms, the LINCS algorithm (57) was used. The six-step equilibration protocol was used for longer and more efficient equilibrations by slowly reducing the position and dihedral angle restraints on protein and lipid molecules. The system temperature was maintained at 37°C using the Nose-Hoover temperature (58) coupling algorithm with a time constant of 1 ps (τ_T = 1 ps) in the canonical ensemble (NVT). Further, the simulation condition was changed from NVT to the isothermal-isobaric (NPT) ensemble to attain appropriate density. The Parrinello-Rahman semi-isotropic pressure coupling (59) was used to maintain the pressure of 1 bar with a time constant (τ_T) of 5 ps and compressibility of 4.5×10^{-5}/bar. Each system is well equilibrated for a total of 16 ns, and the production run was carried out for 1 μs. The trajectory was visualized by using visual molecular dynamics software (60). The last 100 ns trajectory from the production run was used for analysis.

The acyl chain order parameter (S_{CD}) for both the *sn*-1 and *sn*-2 chains was calculated by *gmx order* utility by using the equation

$$S_{CD} = \frac{1}{2}\langle 3\cos^2\theta_z - 1\rangle, \quad (1)$$

where θ_z is the angle between the bilayer normal (z axis) and C-H-bond at each carbon. The headgroup orientation (P-N vector) with respect to the bilayer normal and tilt angle of helices with respect to bilayer normal was calculated using the *gmx gangle* program in GROMACS.

The bending modulus of bilayers was computed based on the Helfrich and Canham theory from the spectrum of undulations in the membrane along a normal direction (61,62). In this method, bending modulus is computed from the variation in the height of the membrane surface above some reference plane. The power spectrum of undulations ($S_u(q)$) is predicted by the expression,

$$S_u(q) = \frac{k_B T}{k_c q^4 + \gamma q^2}, \quad (2)$$

where k_B is Boltzmann's constant, T is the absolute temperature, k_c is the bending rigidity, q is the wavenumber, and γ is the surface tension.

At negligible surface tension, (2) simplifies to:

$$S_u(q) = \frac{k_B T}{k_c q^4}. \quad (3)$$

RESULTS

To evaluate the effect of membrane thickness on the functional properties of GlpG, we first compared the thickness of DLPC-d_{46} and DMPCd_{54} membranes at varying cholesterol concentration using ^{2}H NMR spectroscopy. MLVs with a cholesterol concentration from 0 to 25 mol % were prepared in the absence and presence of GlpG at a molar lipid/GlpG ratio of 125:1. The precise lipid/GlpG ratios were determined using HPTLC and UV absorption measurements (Table S1). Fig. 1 displays some typical ^{2}H NMR spectra of DLPC-d_{46} and DMPC-d_{54} with increasing amounts of cholesterol in the presence and absence of GlpG (all ^{2}H NMR spectra of this study are shown in Fig. S1). All ^{2}H NMR spectra are indicative of fluid liquid-crystalline membranes of lipids undergoing axially symmetric rotational diffusion. The ^{2}H NMR spectra gradually broaden with increasing cholesterol content indicating higher chain order (39). The spectral features observed at higher cholesterol concentration are in agreement with the formation of a liquid-ordered phase as typical for cholesterol-saturated PLs membranes (63). For DMPC-d_{54} membranes in the presence of >20 mol % cholesterol, phase separation into a more ordered and a more disordered phase is observed. In the presence of GlpG, the ^{2}H NMR spectra show some line broadening due to faster T_2 relaxation. We also acquired ^{31}P NMR spectra (Fig. 1) and observed line shapes in agreement with the lamellar liquid crystalline phase state of the membrane with minor isotropic contributions of an isotropic arrangement (<2%). Close inspection of the ^{31}P NMR spectra in the presence of GlpG reveal a second axially symmetric powder pattern (vide infra).

From the ^{2}H NMR spectra, smoothed chain order parameters were calculated (48). For illustration, we plot the order parameters for three different cholesterol concentrations in the presence and in the absence of GlpG in Fig. 2 *A*. It should be noted that, although the vesicles contained a well-defined amount of cholesterol, reconstitution of GlpG from DDM micelles and detergent removal via biobeads changed the cholesterol content, and the exact amount as determined by

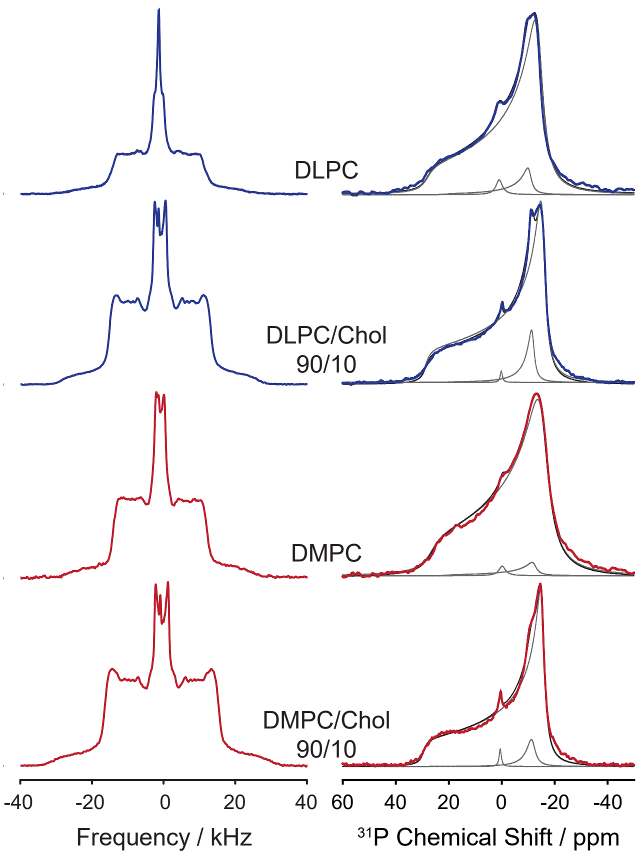

FIGURE 1 Typical ^{2}H (*left*) and ^{31}P NMR (*right*) spectra of DLPC-d_{46} and DMPC-d_{54} membranes in the absence and presence of 10 mol % cholesterol and in the presence of GlpG. The NMR spectra were accumulated for MLVs of chain perdeuterated DLPC-d_{46} or DMPC-d_{54}. ^{31}P NMR spectra were simulated using a superposition of two powder patterns for axially symmetric reorientation ($\Delta\sigma_1 = 42$–45 ppm and $\Delta\sigma_2 = 32$–34 ppm) and a small isotropic line. Best fit simulations of the ^{31}P NMR spectra are shown in black and the individual contributions to the fit are depicted in gray. Measurements were carried out for MLVs at excess acetate buffer at pH 4 and a temperature of 37°C. The cholesterol-free spectra are reproduced from (17).

HPTLC is also given in Fig. 2 *A*. Thus, the individual panels do not exactly show identical cholesterol concentration in the presence and absence of GlpG. As well expected, order parameters of both DLPC-d_{46} and DMPC-d_{54} increased approximately linearly as a function of cholesterol (64). The presence of GlpG has a significant impact on DLPC-d_{46}/cholesterol membranes. In the absence and at low cholesterol content, GlpG orders DLPC-d_{46} membranes slightly, while at high cholesterol content, GlpG decreases the acyl chain order. In contrast, in DMPC-d_{54} membranes in the absence and presence of cholesterol, lipid chain order remains by and large independent of the presence or absence of GlpG.

As membrane order determines membrane thickness, we used Brown's mean-torque model (49) to calculate the projected acyl chain length (Lc) from these order parameters. This is related to hydrophobic thickness but not the only parameter that influences protein function. To account for uncertainties in the cholesterol concentration after protein

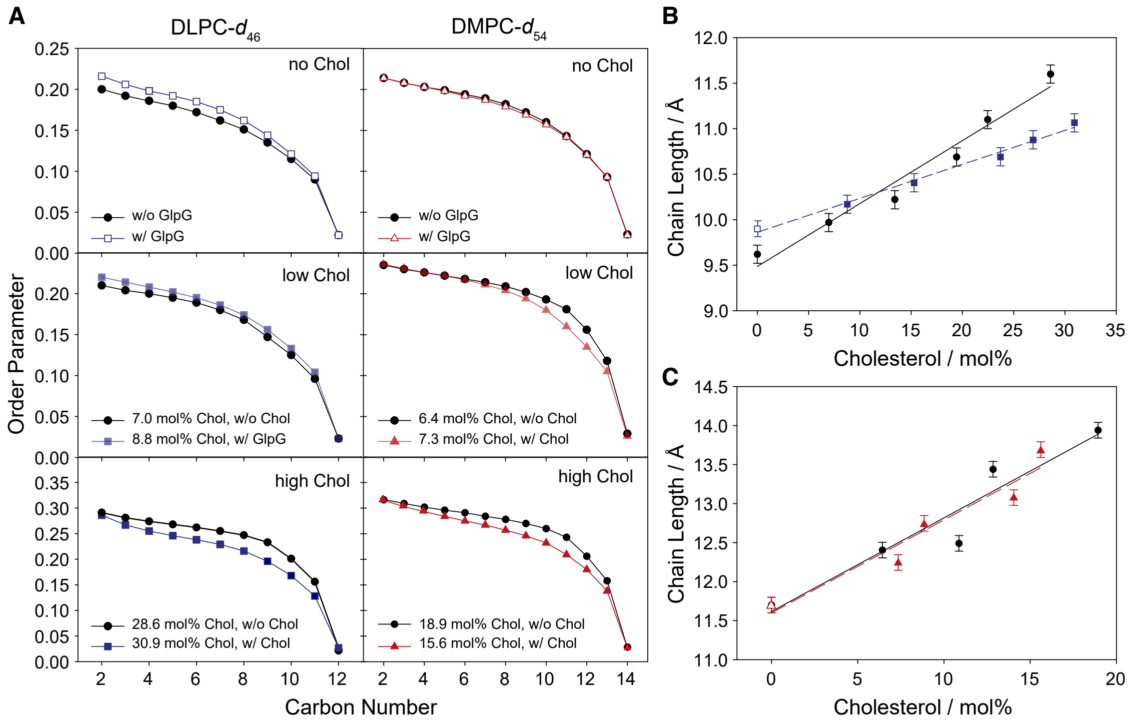

FIGURE 2 Influence of cholesterol on the lipid chain order parameters (*A*) and the projected acyl chain length of DLPC-d_{46} (*B*) and DMPC-d_{54} (*C*) membranes in the absence and presence of GlpG. (*A*) Exemplary order parameter profiles for different cholesterol concentrations in the membrane in the absence and presence of GlpG. Vesicles of low (5 mol %) and high (25 mol % DLPC-d_{46}, 20 mol % DMPC-d_{54}) cholesterol content were prepared. After GlpG reconstitution requiring biobead treatment, the real cholesterol content was measured and is reported in each panel. The projected acyl chain lengths were calculated from the ^{2}H NMR spectra of DLPC-d_{46} (*B*) and DMPC-d_{54} (*C*) membranes at various cholesterol concentrations in the absence (*black circles*) and presence of GlpG (*squares* or *triangles*). The exact cholesterol concentration of each sample was determined from quantitative HPTLC. Lines represent linear regressions. Data for samples that showed phase separation (DMPC-d_{54} with a cholesterol content >20 mol %) were excluded because phase separation had occurred (cf. Fig. S1). The error bars represent the estimated $\pm$0.1 Å variation determined for ^{2}H NMR (65). Measurements were carried out at 37°C, at excess hydration with acetate buffer (pH 4).

reconstitution, both phospholipid and cholesterol concentrations were quantified using HPTLC after conducting the measurements. The projected chain lengths of all preparations as a function of the measured cholesterol concentrations are plotted in Fig. 2 *B*. In the absence of GlpG, the chain length of DLPC-d_{46} increases from 9.7 to 11.6 Å as the cholesterol content is increased from 0 to 30 mol %. In the presence of GlpG, the chain length of cholesterol-free DLPC-d_{46} membranes is slightly increased to 9.9 Å. Such a small thickening effect of GlpG in DLPC-d_{46} membranes has already been found previously (17). In the presence of GlpG, the DLPC-d_{46} chain length increases with increasing amounts of cholesterol, albeit to a smaller extent. At around 31 mol % cholesterol, a chain length of 11.1 Å is estimated. The cholesterol-induced chain length extension follows a linear behavior both in the presence and absence of GlpG (Fig. 2 *B*). Overall, lipid condensation induced by cholesterol is more pronounced in GlpG-free membranes, and condensation is attenuated in the presence of GlpG. This shows that GlpG actively thins DLPC membranes at higher cholesterol content.

A somewhat different picture is obtained for DMPC-d_{54} membranes, as illustrated in Fig. 2 *C* where the projected chain lengths of DMPC-d_{54} are plotted. DMPC-d_{54} acyl chain length is increasing with increasing cholesterol concentration as expected but, within experimental error, no differences are observed in the presence of GlpG. In the cholesterol-free bilayers without GlpG, a chain length of 11.7 Å is measured, which is similar to the chain length of DLPC-d_{46} at the highest measured cholesterol concentration. Upon increasing cholesterol concentration to 19 mol % the chain length of DMPC-d_{54} extends to 13.9 Å. At higher cholesterol concentration, phase separation was observed in the ^{2}H NMR spectra and therefore no chain length was determined (cf. Fig. S1). We previously observed identical chain lengths in DMPCd_{54} bilayers in the absence and presence of GlpG without cholesterol (17). But it is very interesting that GlpG does not thin DMPC-d_{54} membranes of increased hydrophobic thickness induced by increasing the cholesterol content.

Obviously, DLPC-d_{46} and DMPC-d_{54} membranes in the presence of low to moderate cholesterol concentrations respond differently to GlpG. DLPC-d_{46} bilayers decrease their chain length in response to GlpG for cholesterol concentrations exceeding 10 mol %. This effect is not observed for DMPC-d_{54} bilayers, where no difference in membrane

thickness is observed in the measured cholesterol concentration range up to 15 mol %.

To understand how membrane thickness influences the function of GlpG, an assay was used to measure the substrate cleavage kinetics of GlpG (66). The membrane-embedded α-helical model substrate LacYTM2 with the FRET pair EDANS and DABCYL conjugated at either side of the cleavage site was coreconstituted into the GlpG-containing membranes at the preparation stage. Samples were prepared at pH 4 where GlpG is inactive (67). Increasing the pH to 7 activates GlpG and initiates the cleavage reaction. Upon cleavage, the two chromophores are separated by shedding of the N-terminal part from the membrane, indicated by an exponential increase in EDANS fluorescence. A single exponential fit of the fluorescence increase provides a characteristic time constant τ for the enzyme reaction in the respective membrane.

Fig. 3 shows a plot of the characteristic time of the cleavage reaction as a function of leaflet thickness in the respective membranes. Due to small variations in the cholesterol concentration between the preparations for the ^{2}H NMR experiments and the GlpG activity measurements, the linear regression of the data in Fig. 2 was used to relate the exact cholesterol content to the leaflet thickness of the respective samples. To facilitate the comparison, the τ value was normalized to 1 for the DLPC/GlpG preparation in the absence of cholesterol and used as reference (τ_0). This preparation would also represent the thinnest membrane within the series. Fig. 3 reveals that there is no obvious correlation between τ and L_c. With a very small increase in acyl chain length from 9.9 to 10.1 Å the τ value doubles. If the leaflet thickness increases further (>10.4 Å), the τ value drops to 0.8 τ_0 and continues to decrease slightly to a relative cleavage rate of ~0.5 τ_0 at the maximal membrane thickness of 13.5 Å. Obviously, using cholesterol to increase the thickness of saturated membranes changes GlpG function in a highly nonlinear fashion, suggesting that the hydrophobic thickness of the membrane is not the only determinant of GlpG activity. The fastest substrate cleaving reaction is observed for membranes with a hydrophobic thickness in the range of 21–27 Å (i.e., acyl chain lengths of 10.5–13.5 Å). This value corresponds to DLPC membranes at high cholesterol content and DMPC membranes at all cholesterol concentrations investigated here. The specific decrease in GlpG cleavage velocity at low and moderate cholesterol concentrations is also obvious when plotting τ/τ_0 as a function of cholesterol concentration (Fig. S2). It is striking that the enzyme kinetics are significantly influenced by the individual cholesterol concentrations in DLPC membranes but hardly affected in DMPC membranes. Hence, for the enzyme's function, both the precise acyl chain length and the distinctive PL/cholesterol interaction and membrane phase must be considered.

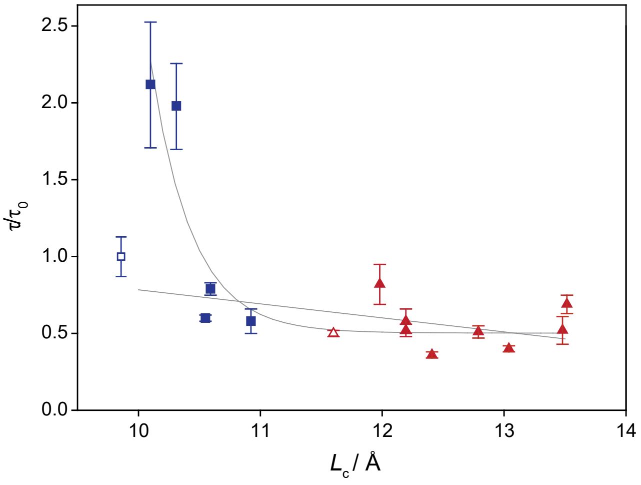

FIGURE 3 Effect of the membrane leaflet thickness on the reaction velocity of GlpG-catalyzed LacYTM2 cleavage in DLPC (*blue squares*) and DMPC (*red triangles*) membranes at varying cholesterol concentrations. DLPC and DMPC samples in the absence of cholesterol are shown as open symbols. The characteristic time constant τ that describes the cleavage reaction was calculated from the cleavage assay using LacYTM2 model substrate (Table S4) and plotted against the projected acyl chain lengths. τ_0 denotes the characteristic cleavage time constant of GlpG in DLPC in the absence of cholesterol as reference (*light blue square*). Projected acyl chain lengths were estimated by taking the determined cholesterol concentration for each sample measured with TLC and using the linear regression fit of the projected chain lengths of DLPC (*blue squares*) and DMPC (*red triangles*) as shown in Fig. 2. The open symbols indicate GlpG cleavage in membranes in the absence of cholesterol. The τ value was normalized to the thinnest membrane DLPC with 0 mol % cholesterol. All measurements were taken at 37°C in multilamellar lipid/protein vesicles. Error bars represent the standard error of the mean ($n \geq 3$).

Given the complicated dependence of τ on the membrane thickness, one could imagine two scenarios for simplification: 1) if we disregard the two outliers of DLPC at low cholesterol concentration, one could see a continuous decrease in cleavage kinetics of GlpG with increasing membrane thickness. If we carry out a linear regression (excluding these two outliers) a weak linear correlation is found with an R^2 of 0.34. Alternatively, we could 2) argue that membrane thickness strongly influences GlpG activity by disregarding the first data point (i.e., GlpG in DLPC membranes in the absence of cholesterol) and fit the data to a single exponential decay giving an R^2 of 0.84. Both fitting results are shown in Fig. 3.

As both scenarios are completely arbitrary and exclusion of data points from the fit has no scientific motivation, we have to consider that GlpG may react in a specific manner to low cholesterol concentrations in DLPC membranes but not in DMPC bilayers. To follow up on this idea, we tried to relate the observed differences in GlpG cleavage efficiency in DLPC membranes at low cholesterol concentrations to structural alterations in the protein. To further explore this option, we carried out MD simulations of GlpG in DLPC membranes at low (5 mol %), intermediate (16 mol %), and high (30 mol %) as well as in DMPC at low (6 mol %), intermediate (15 mol %), and high (23 mol %) cholesterol concentrations. The MD boxes

contained 1 GlpG molecule, and 125 lipids and trajectories of 1 μs for each system were accumulated. Details of the MD setup are given in Table S2.

We first analyzed the GlpG structure in the MD simulations in the different membrane environments. A previous crystal structure of GlpG described an open-cap conformation of GlpG where the capping loop L5 is lifted exposing the previously buried active center of Ser_{201} and two conserved side chains, His_{150} and Asn_{154} (68). In this L5 open conformation, the sidechain of His_{150}, which was suggested to play a role in GlpG function (68), moves closer to Ser_{201}. We calculated the radial distribution function between Ser_{201} and His_{150} for the simulations and found an interesting situation for DLPC at low cholesterol concentration compared with the other membrane environments as shown in Fig. 4. While the radial distribution functions (RDFs) for GlpG in DLPC membranes at high and DMPC membranes at both low and high cholesterol concentration are very similar with a maximum at ~5 Å, the RDF of GlpG in DLPC membranes at low cholesterol concentration is biased toward longer distances with a maximum at around 8 Å. At intermediate cholesterol concentration, RDFs for both lipids are similar to high cholesterol. So, obviously, in a DLPC membrane at low cholesterol concentration, the conformation of GlpG differs from the other three environments. We also observed some alterations in the secondary structure of GlpG in response to the membrane environment. Residues that are mainly exposed to the water surface showed pronounced secondary structure changes (Fig. S3). We corroborated this view by looking at the tertiary structure of GlpG in both membranes at high and low cholesterol. To this end, we calculated the tilt angles of all transmembrane helices (Fig. S4). It is observed that all helices of GlpG are subject to dynamic reorientation. Systematically, the tilt angle of helix 4 (with the active center Ser_{201}), helix 5, and helix 6 is increased in DLPC membranes at low cholesterol content. These conditions are obviously suboptimal for efficient proteolysis.

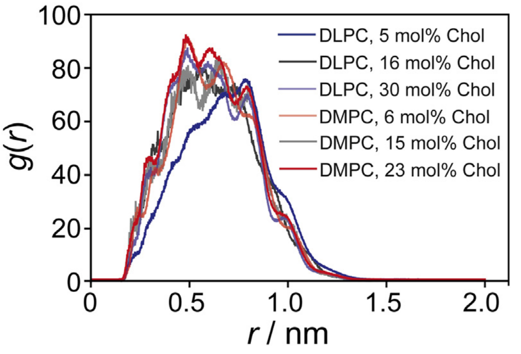

FIGURE 4 Radial distribution function of the distance between Ser_{201} and His_{150} of GlpG in DLPC and DMPC membranes at low, intermediate, and high cholesterol concentration. The functions were calculated from the MD trajectory as described in the text.

Although the ^{2}H NMR spectra of all membrane preparations did not indicate any unusual membrane perturbations induced by either GlpG or cholesterol, previous studies have shown that GlpG can very specifically interact with some lipid species, as indicated in ^{31}P NMR spectra (17). We carried out stationary ^{31}P NMR measurements for DLPC and DMPC membranes at different cholesterol concentrations (^{31}P NMR spectra shown in Figs. 1 and S5). All samples exhibited spectra corresponding to a lamellar bilayer phase along with a very small isotropic contribution in the GlpG-containing membranes (Fig. 1). Interestingly, in the cholesterol-containing DLPC membrane in the presence of GlpG, in addition to the spectral line shape that represents the lamellar phase with $\Delta\sigma_1 = 44.3$ ppm, a second axially symmetric contribution to the NMR spectrum was detected that could be fitted to a CSA tensor span of $\Delta\sigma_2 = 32.6$ ppm. This second contribution with the smaller CSA amounted to approximately 8% of the peak integral. Similar alterations of the ^{31}P NMR spectra were reported earlier for PE/PG 3:1 molar membranes (17). In the PE/PG membranes, the narrow powder pattern contributed approximately 33% and a larger isotropic contribution was also observed (17). A very close inspection of the ^{31}P NMR spectra of DMPC/cholesterol membranes in the presence of GlpG also reveals such a second lamellar contribution with smaller CSA. In fact, all NMR spectra could be simulated using the combination of three line shapes with the parameters $\Delta\sigma_1 = 42$–48 ppm (91–96%), $\Delta\sigma_2 = 32$–34 ppm (3–6%), and an isotropic contribution of 1–2%.

To understand these interesting observations, we returned to the MD simulations for the four different membrane systems. NMR is an ensemble technique and cannot distinguish molecules of the same kind in different environments unless they are in slow exchange, as seen in the ^{31}P NMR spectra. In NMR, slow exchange refers to the situation when two different conformations give different NMR spectra and the measured spectrum represents a superposition of both subspectra of the individual states and the integrals are proportional to the population of each state. On the other hand, the MD can precisely differentiate between annular and bulk lipids. Therefore, we analyzed the order parameters of the annular versus bulk lipids. Depending on cholesterol content, the number of annular lipids varies between 22 and 33 (Table S3). Histograms of the distribution of annular lipids (cholesterol versus PC) are shown in Fig. S6. The lipid annulus contained only phospholipids and no cholesterol in DLPC or DMPC membranes at low cholesterol. At intermediate concentration, the lipid annulus around GlpG in DLPC contained 3.6% cholesterol (vs. 19.6% in the bulk). In DMPC we determined a cholesterol content of 7.1% (vs. 17.5% in the bulk). At high cholesterol, the annular layer of GlpG in DLPC contained 18% cholesterol (vs. 33.3% in the bulk) or 10.3% in DMPC, where the bulk cholesterol concentration was 27%. This suggests that GlpG prefers contact to phospholipids and avoids cholesterol.

Having identified annular lipids in the MD simulations, the next step was to calculate their chain order parameters, which are shown in Fig. 5, *A* and *B* for the *sn*-1 and in Fig. S7 for the *sn*-2 chains in comparison with the bulk lipid chain order parameters. Order parameters are similar for the annular and bulk DLPC lipids at low cholesterol content (Fig. 5 *A*). Annular DMPC lipids at low cholesterol concentration show a bit higher order in the upper and a bit lower order in the lower half of the chains (Fig. 5 *B*). However, at high cholesterol concentration, both DLPC and DMPC annular lipids exhibit much lower order parameters than the bulk lipids. Similar results are obtained for the *sn*-2 chains (Fig. S7). From these data we calculated the chain length (L_c) for the annular lipids from the average of the *sn*-1 and *sn*-2 chains (as done with the experimental data) using the mean torque model and compare them with the order parameters for the bulk lipids calculated from MD simulations in the absence of GlpG. A plot of L_c versus cholesterol concentration for the annular and bulk lipids is shown in Fig. 5 *C*.

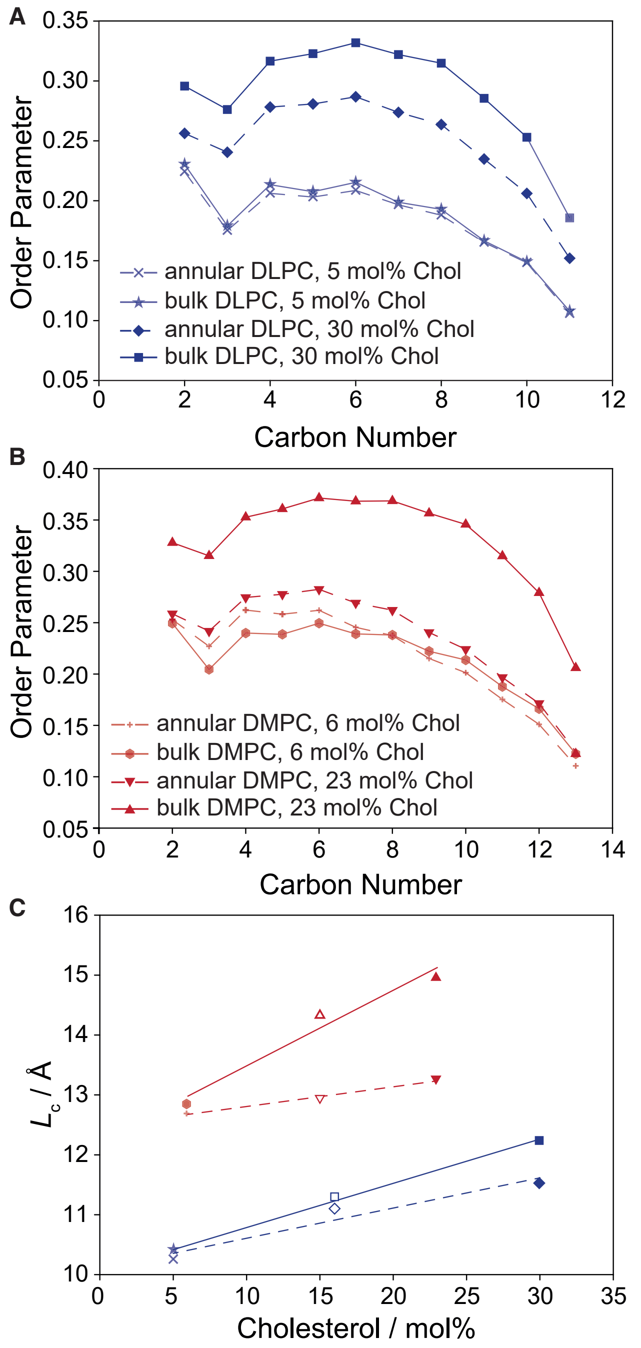

FIGURE 5 Order parameters (*A* and *B*) and average projected chain lengths (*C*) of annular (*dashed lines*) and bulk lipids (*solid line*) for DLPC (*blue*) and DMPC (*red*) membranes in the presence of GlpG. (*A* and *B*) Order parameters for the *sn*-1 chain of DLPC (*blue*) and DMPC (*red*) calculated from the MD simulation. Order parameter plots for the *sn*-2 chains are shown in Fig. S7. (*C*) Average projected chain lengths for the annular (*dashed lines*) and bulk lipids (*solid lines*) calculated from the MD simulations at high and low cholesterol concentrations. The chain lengths are determined from the average in chain order for the *sn*-1 and the *sn*-2 chains.

The observations from this comparison are summarized as follows: 1) at low cholesterol content, there is no clear difference between the order/acyl chain length of annular and bulk lipids. 2) Chain order/length increases for both annular and bulk lipids at high cholesterol concentration. 3) Annular lipids are more disordered than bulk lipids at high cholesterol concentration. 4) The difference in chain order/chain length between annular and bulk lipids is more pronounced in DMPC at high cholesterol compared with DLPC membranes.

We also calculated the bending elasticity k_c of the different membranes from the MD simulations using the Helfrich and Canham theory (61,62). Values are given in Fig. 6. The values calculated agree relatively well with experimental data (69,70) and values from other MD simulations (71). For DLPC, increasing cholesterol concentration slightly increases the bending modulus but, within the error, no alteration through the presence of GlpG is observed. For DMPC, the increase in k_c upon increase in cholesterol concentration is very pronounced and also higher bending moduli are observed in the presence of GlpG, especially at high cholesterol content.

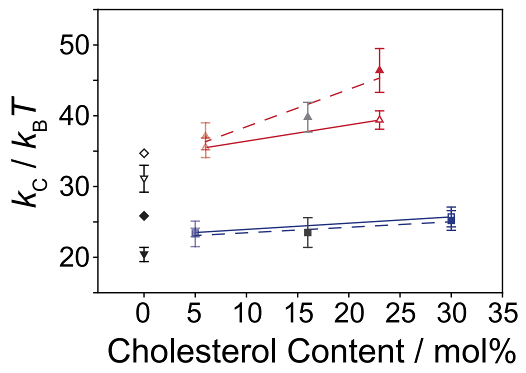

FIGURE 6 Bending elasticity k_c (in units of $k_B T$) of DLPC (*blue*) and DMPC (*red*) membranes in the absence (*open symbols*) and presence of GlpG (*filled symbols*). Values are calculated from MD simulations using the Helfrich and Canham theory (61,62). For comparison, literature values for k_c are given in the absence of GlpG and cholesterol determined from experiment (*upside down triangle*, (69,70)) or MD simulations (*diamonds*, (71)). Error bars represent SD with $n = 2$.

The MD data also allow to analyze the PC headgroup orientation for the annular and bulk lipids individually. The average headgroup orientation and dynamics determines the width of the CSA tensor observed in the ^{31}P NMR powder spectra. From the MD, we determined the average orientation of the P-N headgroup dipole relative to the membrane normal and observed a narrow distribution around 90° for the bulk lipids as shown in Fig. 7. A molecular sketch of how the headgroup orientation is defined is shown in Fig. S8. For the annular lipids, the histograms of the same bond vector are significantly broader. For DMPC at high cholesterol, the distribution is also shifted to higher angles. This suggests that the annular lipids sample a larger distribution of angles leading to further averaging of the CSA tensor, giving rise to the second powder pattern with a narrower span of the CSA tensor ($\Delta\sigma_2$, cf. Fig. 1). The exchange between annular and bulk lipids must be slow (i.e., milliseconds) to observe the spectral characteristics of the two distinct states. On the other hand, we would also expect to observe these two distinct states in the ^{2}H NMR spectra. Clear indication for phase separation was only given in ^{2}H NMR spectra at cholesterol content exceeding 20 mol % (cf. Fig. S1). At lower cholesterol concentration, the resolution of the ^{2}H NMR spectra of the fully chain perdeuterated lipids is not sufficient to provide a clear answer, the narrowest Pake doublet stemming from the terminal methyl group does not indicate the presence of two phases. This suggests that the impact of GlpG on the annular lipid is predominantly exerted through headgroup interaction with GlpG.

DISCUSSION

We aimed to quantify the effect of the hydrophobic thickness of the host membrane on the catalytic activity of GlpG that was previously found in membranes composed of different lipid species varying in their headgroup and chain structure (17). To keep things simple and comparable, we used fully saturated DLPC and DMPC lipids to build the host membrane. Although not very physiological, these two lipids only vary in their acyl chain length, but otherwise feature exactly the same chemical characteristics. To alter membrane thickness, we added cholesterol, which is very well known for its condensing effect, leading to a linear increase in membrane thickness as a function of cholesterol content (72,73). Thus, using varying cholesterol concentrations, membrane thickness can be adjusted with good resolution. Although a very suitable tool to alter the bulk properties of the host membrane for GlpG, some caveats of our strategy have to be discussed. First, both saturated phosphatidylcholine and cholesterol lipids are not relevant for the plasma membrane of *E. coli*, which is the host organism of GlpG. Although GlpG functions well in DLPC and DMPC membranes (17), they represent an artificial environment for the *E. coli* protease. Second, adding cholesterol may induce very specific effects that are of biophysical importance for membrane proteins (74,75), but not relevant as *E. coli* membranes do not contain cholesterol or any other sterol. It is well studied that cholesterol represents an important lipid and even an allosteric modulator of membrane proteins (74,75). The anomalies we observed in GlpG function in DLPC membranes at low cholesterol concentration may support the general importance of the sterol for α-helical membrane proteins but has no direct relevance for *E. coli* proteins.

Most importantly, third, cholesterol not only alters membrane thickness but also impacts the elastic properties of the bilayer and modulates other effects (44,45). Considering such a complex spectrum of properties, it is very challenging to establish a straightforward relationship between membrane protein activity and a single lipid membrane

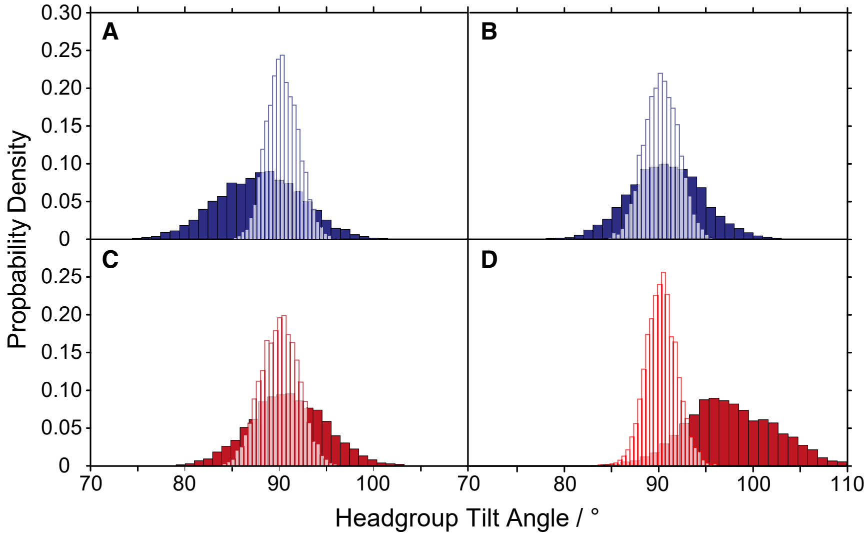

FIGURE 7 Histograms for the orientations of the P-N headgroup dipole of the annular (*filled bars*) and bulk lipids (*white bars*) for DLPC (*blue*) and DMPC membranes (*red*) at low (*A* and *C*) and high cholesterol content (*B* and *D*). Histograms were obtained from MD simulations.

property at varying cholesterol concentrations. In addition to allosteric effects, issues such as bending rigidity, curvature energy, and other physical alterations need be considered. It has been elegantly shown that lipid fluctuations, highly impacted by cholesterol, increase bending rigidity of membranes (44). Work on small membrane-embedded peptides has also demonstrated that membrane bending rigidity is not altered by the peptide (76), but little is known how membrane bending rigidity influences membrane protein activity. Our data show that low cholesterol concentration slows down GlpG cleavage velocity in DLPC membranes (Fig. 3), while membrane elasticity remains constant (Fig. 6). In contrast, varying cholesterol concentrations in DMPC membranes did not alter GlpG activity much (Fig. 3), but the presence of the protein increased k_c significantly (Fig. 6).

In discussing the impact of cholesterol on lipid membranes, usually the individual parameters that are influenced are considered as more or less decoupled. When it comes to pure lipid membranes, this approach is useful as long as lipid molecules are homogeneously distributed. In heterogeneous membranes, featuring domains or embedded proteins, the distinction between individual parameters becomes difficult and a more integrative approach needs to be taken. This is observed here, for instance, we see that GlpG alters the thickness of the host DLPC membrane, which has an influence on protein function (Fig. 3), but the bending elasticity of the membrane is uninfluenced (Fig. 6). In contrast, in DMPC membranes, the presence of GlpG does not influence membrane thickness, but bending elasticity is strongly modulated. So, clearly, properties, such as chain order, elasticity, curvature, hydrophobic thickness, etc., are highly coupled in the presence of heterogeneities and act synergistically, requiring an individual treatment of each specific condition. This also calls for more unifying models and concepts of lipid-protein interaction. Alternative ideas and models such as the dynamic landscape of lipid membranes may be helpful in this context (93). One observation that agrees for all investigated model systems is that GlpG avoids contact with cholesterol as the annular lipid layer is highly depleted in this sterol (Table S3).

Coming back to hydrophobic matching, protein aggregation is also a way of avoiding unfavorable hydrophobic mismatch. Although protein oligomerization was originally proposed as a regulatory mechanism for GlpG (77), more recent work has challenged this concept (78). Of course, one can never exclude an in vitro effect related to the high packing density of protein in our samples. While cellular membranes are very densely packed with protein, reconstitution artifacts may lead to unphysiological aggregation of protein. Under all conditions used in this study, GlpG was always fully functional, which excludes that unspecific protein aggregation occurred.

The very specific impact of the lipid matrix on protein function is shown here in the very simple lipid composition. While we can confirm the previous conclusion that GlpG function is related to hydrophobic matching, the window of membrane thicknesses for efficient proteolysis is relatively broad. As discussed above, GlpG function is not only determined by hydrophobic matching alone. This is already seen in the difference in the interaction of GlpG with either DLPC or DMPC membranes: while DLPC membranes in the presence of cholesterol are actively altered, no such effect was observed for DMPC bilayers (cf. Fig. 2). Surprisingly, at certain molar ratios of DLPC and cholesterol, GlpG function is significantly impaired and catalysis is more than a factor of 2 slower. This suggests that there is a rather specific effect of DLPC membranes in the presence of low cholesterol concentration, which leads to alterations in GlpG structure affecting catalysis. The exact nature of this remarkable effect is currently unclear. While the impact of cholesterol on pure DMPC membranes, their thickness, bending rigidity, etc., is relatively well understood (79–82), few data are available for DLPC.

While our initial intension to understand the impact of a simple physical parameter on membrane protein function in very idealistic membrane mimetics of two components only provided rather controversial results, we have to recall that mammalian cell membranes not only consist of hundreds of different lipid species but that these bilayers feature a unique and dynamic lateral organization and dynamic fluctuations in lipid distribution (22). Membrane proteins can sense alterations in local curvature, changes in the electrostatics, or the elastic properties of the direct lipid environment. Even the simple two-lipid system employed here already shows that the annular shell of GlpG exhibits changes in cholesterol distribution compared with the bulk and alterations in headgroup tilt and chain order (Figs. 6, S6, and S7; Table S3). As cholesterol is known to increase the thickness of membranes, the effect of cholesterol depletion in the vicinity of GlpG may also contribute to the thinning of the annular layer in DLPC. Previous work has also shown that cholesterol spans the bilayer midplane in DLPC but not in DMPC membranes and assumes a larger tilt angle (83). These differences must be considered when looking at GlpG function in each membrane environment. In addition, cholesterol-induced lipid condensation cannot be separated from the effect of cholesterol on membrane bending rigidity, which increases with increasing cholesterol concentration (44) leading to a decrease in fluidity.

Bending rigidity is a bulk property of the membrane and cannot be defined for the annular lipid layer. So considering the impact of bending rigidity on protein function also requires considering the impact of a membrane protein on bending rigidity. Alternatively, we looked at differences between annular and bulk lipids observable from the MD trajectories. This analysis revealed that properties of annular and bulk lipids are rather different caused by an active alteration of the membrane properties by the protein. This complicated interplay of lipid-protein interaction may also

represent a means by which the biological activity of proteins is regulated upon alteration of lipid environment in the vicinity of a protein (3,84). Importantly, by MD simulation we observed coupling of the bilayer dimension to the tilt angles of the α helices within the helical bundle of GlpG's rhomboid fold. Of note, a conserved GlyxxxGly helix dimerization motif in transmembrane domain 6 in the core of the rhomboid domain is essential for a variety of different rhomboid family proteins (85–88). Therefore, it is tempting to speculate that the reversible dynamic alteration of the rhomboid domain in response to varying bilayer properties is a physiologically relevant regulatory mechanism. Consistent with this idea, the activity of mammalian rhomboid protease RHBDL4 has been shown to respond to the membrane cholesterol level (89). Not to be underestimated, all cellular membranes consist of a multitude of different lipids and are often characterized by their asymmetric distribution between the inner and outer leaflet, which is completely disregarded in most biophysical studies, but also determines protein function (19).

CONCLUSIONS

In conclusion, an optimal membrane environment seems to be an important requirement for rhomboid-catalyzed intramembrane proteolysis. This is related to the hydrophobic thickness of the membrane but this is not the only parameter that influences protein function. However, as observed experimentally, using cholesterol as a modulator of hydrophobic thickness for DLPC and DMPC membranes brings about a complex interplay of the lipids with the model rhomboid protease GlpG. While DLPC/cholesterol membranes are thinned by GlpG, no such active intervention was observed in DMPC/cholesterol membranes. MD simulations confirmed that the annular lipid layer of saturated PC/cholesterol membranes is strongly modified by interactions with the protein. Some DLPC/cholesterol ratios alter the structure of GlpG resulting in reduced cleavage activity. It is well studied that cholesterol can have several membrane effects, especially the condensing effect on lipid acyl chains responsible for membrane thickening, but also alterations in its tilt angle and membrane insertion depth, especially in thin membranes (83), and it can create phase separation (90). Consequently, cholesterol is a known modulator of protein function (75,91,92). Therefore, separating the functional impact of cholesterol on GlpG function and the impact of cholesterol on the bilayer properties is rather complex. Generally, GlpG seems relatively robust against quite drastic modulation of hydrophobic thickness and cleaves substrates rather efficiently in all membranes investigated. Alterations in cleavage efficiency can be related to small changes in the lipid environment of the protease, leading to moderate alterations in GlpG structure, which may provide a mechanism by which proteolytic activity is controlled. But it seems that no universal effect of bilayer properties on GlpG can be derived. While cholesterol, DLPC, or DMPC are not naturally found in bacteria, eukaryotic rhomboid pseudoproteases are present in very diverse membranes such as mitochondria and the endoplasmic reticulum, which could also be as thin and unstable as the DLPC/cholesterol membranes. Our data suggest that even abundant bulk lipids show some modulatory effects on GlpG and possibly rhomboids in general. Thus, it will be intriguing to explore the physiological or pathophysiological impact of different membranes and hydrophobic mismatches on the activity and function of a broader range of rhomboids. It becomes more and more evident that the role of the lipid bilayer goes beyond the simple stabilization of protein structure.

ACKNOWLEDGMENTS

D.H. and O.E. acknowledge support from the Deutsche Forschungsgemeinschaft (DFG) project nos. 490761708 and 280771871, and by the European Social Funds (ESF) and the Free State of Saxony (Junior Research Group UniDyn, project no. SAB 100382164). A.V.M. and D.C. acknowledge use of the PARAM UTKARSH HPC facility of the National Supercomputing Mission project and thank the Centre for Cyber Physical Systems (CCPS), National Institute of Technology Karnataka, Surathkal, for the financial support for the same. A.V.M. acknowledges NITK for fellowship.

AUTHOR CONTRIBUTIONS

D.H., D.C., M.K.L., and O.E. planned the research. O.E., V.D., A.V.M., and C.F. performed the experiments. O.E., D.H., V.D., A.V.M., D.C., and C.F. analyzed the data. O.E. and D.H. wrote the article with contributions from all authors.

DECLARATION OF INTERESTS

The authors declare no competing interests.

SUPPORTING MATERIAL

Supporting material can be found online at https://doi.org/10.1016/j.bpj.2024.10.019.

REFERENCES

1. van Meer, G., D. R. Voelker, and G. W. Feigenson. 2008. Membrane lipids: where they are and how they behave. *Nat. Rev. Mol. Cell Biol.* 9:112–124. https://doi.org/10.1038/nrm2330.

2. Harayama, T., and H. Riezman. 2018. Understanding the diversity of membrane lipid composition. *Nat. Rev. Mol. Cell Biol.* 19:281–296. https://doi.org/10.1038/nrm.2017.138.

3. Huster, D., S. Maiti, and A. Herrmann. 2024. Phospholipid membranes as chemically and functionally tunable materials. *Adv. Mater.* 36:e2312898. https://doi.org/10.1002/adma.202312898.

4. Thakur, N., A. P. Ray, ..., M. T. Eddy. 2023. Anionic phospholipids control mechanisms of GPCR-G protein recognition. *Nat. Commun.* 14:794. https://doi.org/10.1038/s41467-023-36425-z.

5. Rohacs, T. 2024. Phosphoinositide Regulation of TRP Channels: A functional overview in the structural era. *Annu. Rev. Physiol.* 86:329–355. https://doi.org/10.1146/annurev-physiol-042022-013956.

6. Yuan, Z., and S. B. Hansen. 2023. Cholesterol regulation of membrane proteins revealed by two-color super-resolution imaging. *Membranes.* 13:250. https://doi.org/10.3390/membranes13020250.

7. Gada, K. D., and D. E. Logothetis. 2022. PKC regulation of ion channels: The involvement of PIP_2. *J. Biol. Chem.* 298:102035. https://doi.org/10.1016/j.jbc.2022.102035.

8. Nyholm, T. K. M. 2015. Lipid-protein interplay and lateral organization in biomembranes. *Chem. Phys. Lipids.* 189:48–55. https://doi.org/10.1016/j.chemphyslip.2015.05.008.

9. Contreras, F. X., A. M. Ernst, …, B. Brügger. 2011. Specificity of intramembrane protein-lipid interactions. *Cold Spring Harbor Perspect. Biol.* 3:a004705. https://doi.org/10.1101/cshperspect.a004705.

10. Killian, J. A., and T. K. M. Nyholm. 2006. Peptides in lipid bilayers: the power of simple models. *Curr. Opin. Struct. Biol.* 16:473–479. https://doi.org/10.1016/j.sbi.2006.06.007.

11. Killian, J. A., and G. von Heijne. 2000. How proteins adapt to a membrane-water interface. *Trends Biochem. Sci.* 25:429–434. https://doi.org/10.1016/s0968-0004(00)01626-1.

12. White, S. H., A. S. Ladokhin, …, K. Hristova. 2001. How membranes shape protein structure. *J. Biol. Chem.* 276:32395–32398. https://doi.org/10.1074/jbc.R100008200.

13. Killian, J. A. 1998. Hydrophobic mismatch between proteins and lipids in membranes. *Biochim. Biophys. Acta.* 1376:401–415. https://doi.org/10.1016/s0304-4157(98)00017-3.

14. de Planque, M. R. R., and J. A. Killian. 2003. Protein-lipid interactions studied with designed transmembrane peptides: role of hydrophobic matching and interfacial anchoring. *Mol. Membr. Biol.* 20:271–284. https://doi.org/10.1080/09687680310001605352.

15. Soubias, O., S. L. Niu, …, K. Gawrisch. 2008. Lipid-rhodopsin hydrophobic mismatch alters rhodopsin helical content. *J. Am. Chem. Soc.* 130:12465–12471. https://doi.org/10.1021/ja803599x.

16. Litman, B. J., and D. C. Mitchell. 1996. A role for phospholipid polyunsaturation in modulating membrane protein function. *Lipids.* 31:S193–S197. https://doi.org/10.1007/BF02637075.

17. Engberg, O., D. Ulbricht, …, D. Huster. 2022. Rhomboid-catalyzed intramembrane proteolysis requires hydrophobic matching with the surrounding lipid bilayer. *Sci. Adv.* 8:eabq8303. https://doi.org/10.1126/sciadv.abq8303.

18. Cybulski, L. E., and D. de Mendoza. 2011. Bilayer hydrophobic thickness and integral membrane protein function. *Curr. Protein Pept. Sci.* 12:760–766. https://doi.org/10.2174/138920311798841681.

19. Piller, P., E. F. Semeraro, …, G. Pabst. 2023. Allosteric modulation of integral protein activity by differential stress in asymmetric membranes. *PNAS Nexus.* 2:pgad126. https://doi.org/10.1093/pnasnexus/pgad126.

20. Soubias, O., A. J. Sodt, …, K. Gawrisch. 2023. Physiological changes in bilayer thickness induced by cholesterol control GPCR rhodopsin function. *Biophys. J.* 122:973–983. https://doi.org/10.1016/j.bpj.2022.11.2937.

21. Mei, N., M. Robinson, …, Z. Leonenko. 2020. Melatonin alters fluid phase coexistence in POPC/DPPC/cholesterol Membranes. *Biophys. J.* 119:2391–2402. https://doi.org/10.1016/j.bpj.2020.10.030.

22. Levental, I., and E. Lyman. 2023. Regulation of membrane protein structure and function by their lipid nano-environment. *Nat. Rev. Mol. Cell Biol.* 24:107–122. https://doi.org/10.1038/s41580-022-00524-4.

23. Sawczyc, H., T. Tatsuta, …, A. Lange. 2024. Lipid-polymer nanoparticles to probe the native-like environment of intramembrane rhomboid protease GlpG and its activity. *Nat. Commun.* 15:7533. https://doi.org/10.1038/s41467-024-51989-0.

24. Wagner, N., M. Gutweiler, …, K. Dose. 1987. Coreconstitution of bacterial ATP synthase with monomeric bacteriorhodopsin into liposomes. A comparison between the efficiency of monomeric bacteriorhodopsin and purple membrane patches in coreconstitution experiments. *Eur. J. Biochem.* 165:177–183. https://doi.org/10.1111/j.1432-1033.1987.tb11209.x.

25. Kreutzberger, A. J. B., M. Ji, …, S. Urban. 2019. Rhomboid distorts lipids to break the viscosity-imposed speed limit of membrane diffusion. *Science.* 363:eaao0076. https://doi.org/10.1126/science.aao0076.

26. Wu, X., M. Siggel, …, T. A. Rapoport. 2020. Structural basis of ER-associated protein degradation mediated by the Hrd1 ubiquitin ligase complex. *Science.* 368:eaaz2449. https://doi.org/10.1126/science.aaz2449.

27. Bondar, A. N., C. del Val, and S. H. White. 2009. Rhomboid protease dynamics and lipid interactions. *Structure.* 17:395–405. https://doi.org/10.1016/j.str.2008.12.017.

28. Vinothkumar, K. R. 2011. Structure of rhomboid protease in a lipid environment. *J. Mol. Biol.* 407:232–247. https://doi.org/10.1016/j.jmb.2011.01.029.

29. Wang, Y., Y. Zhang, and Y. Ha. 2006. Crystal structure of a rhomboid family intramembrane protease. *Nature.* 444:179–180. https://doi.org/10.1038/nature05255.

30. Huster, D., K. Arnold, and K. Gawrisch. 1998. Influence of docosahexaenoic acid and cholesterol on lateral lipid organization in phospholipid mixtures. *Biochemistry.* 37:17299–17308. https://doi.org/10.1021/bi980078g.

31. Brown, M. F. 1994. Modulation of rhodopsin function by properties of the membrane bilayer. *Chem. Phys. Lipids.* 73:159–180. https://doi.org/10.1016/0009-3084(94)90180-5.

32. Botelho, A. V., N. J. Gibson, …, M. F. Brown. 2002. Conformational energetics of rhodopsin modulated by nonlamellar-forming lipids. *Biochemistry.* 41:6354–6368. https://doi.org/10.1021/bi011995g.

33. Kucerka, N., M. P. Nieh, and J. Katsaras. 2011. Fluid phase lipid areas and bilayer thicknesses of commonly used phosphatidylcholines as a function of temperature. *Biochim. Biophys. Acta.* 1808:2761–2771. https://doi.org/10.1016/j.bbamem.2011.07.022.

34. Holte, L. L., S. A. Peter, …, K. Gawrisch. 1995. ^{2}H nuclear magnetic resonance order parameter profiles suggest a change of molecular shape for phosphatidylcholines containing a polyunsaturated acyl chain. *Biophys. J.* 68:2396–2403. https://doi.org/10.1016/S0006-3495(95)80422-4.

35. Lewis, B. A., and D. M. Engelman. 1983. Lipid bilayer thickness varies linearly with acyl chain length in fluid phosphatidylcholine vesicles. *J. Mol. Biol.* 166:211–217. https://doi.org/10.1016/s0022-2836(83)80007-2.

36. Fischer, M., B. Schwarze, …, H. A. Scheidt. 2022. Predicting ^{2}H NMR acyl chain order parameters with graph neural networks. *Comput. Biol. Chem.* 100:107750. https://doi.org/10.1016/j.compbiolchem.2022.107750.

37. Engberg, O., A. Bochicchio, …, D. Huster. 2020. Serotonin alters the phase equilibrium of a ternary mixture of phospholipids and cholesterol. *Front. Physiol.* 11:578868. https://doi.org/10.3389/fphys.2020.578868.

38. Otten, D., M. F. Brown, and K. Beyer. 2000. Softening of membrane bilayers by detergents elucidated by deuterium NMR spectroscopy. *J. Phys. Chem. B.* 104:12119–12129. https://doi.org/10.1021/jp001505e.

39. Huster, D., H. A. Scheidt, …, P. Müller. 2005. Desmosterol may replace cholesterol in lipid membranes. *Biophys. J.* 88:1838–1844. https://doi.org/10.1529/biophysj.104.048926.

40. Soubias, O., and K. Gawrisch. 2012. The role of the lipid matrix for structure and function of the GPCR rhodopsin. *Biochim. Biophys. Acta.* 1818:234–240. https://doi.org/10.1016/j.bbamem.2011.08.034.

41. Soubias, O., O. Saurel, …, A. Milon. 2002. High resolution ^{13}C NMR spectra on oriented lipid bilayers: from quantifying the various sources of line broadening to performing 2D experiments with 0.2-0.3 ppm resolution in the carbon dimension. *J. Biomol. NMR.* 24:15–30. https://doi.org/10.1023/A:1020656611380.

42. Bjornestad, V. A., and R. Lund. 2023. Pathways of membrane solubilization: a structural study of model lipid vesicles exposed to classical detergents. *Langmuir.* 39:3914–3933. https://doi.org/10.1021/acs.langmuir.2c03207.

43. Ali, M. R., K. H. Cheng, and J. Huang. 2007. Assess the nature of cholesterol-lipid interactions through the chemical potential of

cholesterol in phosphatidylcholine bilayers. *Proc. Natl. Acad. Sci. USA*. 104:5372–5377. https://doi.org/10.1073/pnas.0611450104.

44. Doktorova, M., G. Khelashvili, …, M. F. Brown. 2023. Molecular simulations and NMR reveal how lipid fluctuations affect membrane mechanics. *Biophys. J.* 122:984–1002. https://doi.org/10.1016/j.bpj.2022.12.007.
45. Chakraborty, S., M. Doktorova, …, R. Ashkar. 2020. How cholesterol stiffens unsaturated lipid membranes. *Proc. Natl. Acad. Sci. USA*. 117:21896–21905. https://doi.org/10.1073/pnas.2004807117.
46. Hope, M. J., M. B. Bally, …, P. R. Cullis. 1985. Production of large unilamellar vesicles by a rapid extrusion procedure. Characterization of size distribution, trapped volume, and ability to maintain a membrane potential. *Biochim. Biophys. Acta*. 812:55–65. https://doi.org/10.1016/0009-2614(76)80392-2.
47. Davis, J. H., K. R. Jeffrey, …, T. P. Higgs. 1976. Quadrupolar echo deuteron magnetic resonance spectroscopy in ordered hydrocarbon chains. *Chem. Phys. Lett.* 42:390–394. https://doi.org/10.1016/0009-2614(76)80392-2.
48. Lafleur, M., B. Fine, …, M. Bloom. 1989. Smoothed orientational order profile of lipid bilayers by ^{2}H nuclear magnetic resonance. *Biophys. J.* 56:1037–1041. https://doi.org/10.1016/S0006-3495(89)82749-3.
49. Petrache, H. I., S. W. Dodd, and M. F. Brown. 2000. Area per lipid and acyl length distributions in fluid phosphatidylcholines determined by ^{2}H NMR spectroscopy. *Biophys. J.* 79:3172–3192. https://doi.org/10.1016/S0006-3495(00)76551-9.
50. Pott, T., and E. J. Dufourc. 1995. Action of melittin on the DPPC-cholesterol liquid-ordered phase: a solid state ^{2}H and ^{31}P NMR study. *Biophys. J.* 68:965–977. https://doi.org/10.1016/S0006-3495(95)80272-9.
51. Wu, Z., N. Yan, …, Y. Shi. 2006. Structural analysis of a rhomboid family intramembrane protease reveals a gating mechanism for substrate entry. *Nat. Struct. Mol. Biol.* 13:1084–1091. https://doi.org/10.1038/nsmb1179.
52. Jo, S., T. Kim, …, W. Im. 2008. CHARMM-GUI: a web-based graphical user interface for CHARMM. *J. Comput. Chem.* 29:1859–1865. https://doi.org/10.1002/jcc.20945.
53. Abraham, M. J. M. ,T., R. Schulz, …, E. Lindahl. 2015. High performance molecular simulations through multi-level parallelism from laptops to supercomputers. *SoftwareX*. 1–2:19–25. https://doi.org/10.1016/j.softx.2015.06.001.
54. Huang, J., and A. D. MacKerell, Jr. 2013. CHARMM36 all-atom additive protein force field: validation based on comparison to NMR data. *J. Comput. Chem.* 34:2135–2145. https://doi.org/10.1002/jcc.23354.
55. Jorgensen, W. L., J. Chandrasekhar, …, M. L. Klein. 1983. Comparison of simple potential functions for simulating liquid water. *J. Chem. Phys.* 79:926–935. https://doi.org/10.1063/1.445869.
56. Essmann, U., L. Perera, …, L. G. Pedersen. 1995. A smooth particle mesh Ewald method. *J. Chem. Phys.* 103:8577–8593. https://doi.org/10.1063/1.445869.
57. Hess, B., H. Bekker, …, J. G. E. M. Fraaije. 1997. LINCS: A linear constraint solver for molecular simulations. *J. Comput. Chem.* 18:1463–1472. https://doi.org/10.1002/(SICI)1096-987X(199709)18:123.0.CO;2-H.
58. Evans, D. J., and B. L. Holian. 1985. The Nose–Hoover thermostat. *J. Chem. Phys.* 83:4069–4074. https://doi.org/10.1063/1.449071.
59. Nosé, S., and M. L. Klein. 1983. Constant pressure molecular dynamics for molecular systems. *Mol. Phys.* 50:1055–1076. https://doi.org/10.1080/00268978300102851.
60. Humphrey, W., A. Dalke, and K. Schulten. 1996. Visual molecular dynamics. *J. Mol. Graph.* 14:33–38. https://doi.org/10.1016/0263-7855(96)00018-5.
61. Canham, P. B. 1970. The minimum energy of bending as a possible explanation of the biconcave shape of the human red blood cell. *J. Theor. Biol.* 26:61–81. https://doi.org/10.1016/s0022-5193(70)80032-7.
62. Helfrich, W. 1973. Elastic properties of lipid bilayers: theory and possible experiments. *Z. Naturforsch. C Biosci.* 28:693–703. https://doi.org/10.1515/znc-1973-11-1209.
63. Davis, J. H., J. J. Clair, and J. Juhasz. 2009. Phase equilibria in DOPC/DPPC-d_{62}/cholesterol mixtures. *Biophys. J.* 96:521–539. https://doi.org/10.1016/j.bpj.2008.09.042.
64. Scheidt, H. A., P. Müller, …, D. Huster. 2003. The potential of fluorescent and spin-labeled steroid analogs to mimic natural cholesterol. *J. Biol. Chem.* 278:45563–45569. https://doi.org/10.1074/jbc.M303567200.
65. Feller, S. E., K. Gawrisch, and A. D. MacKerell, Jr. 2002. Polyunsaturated fatty acids in lipid bilayers: intrinsic and environmental contributions to their unique physical properties. *J. Am. Chem. Soc.* 124:318–326. https://doi.org/10.1021/ja0118340.
66. Ticha, A., S. Stanchev, …, K. Strisovsky. 2017. Sensitive versatile fluorogenic transmembrane peptide substrates for rhomboid intramembrane proteases. *J. Biol. Chem.* 292:2703–2713. https://doi.org/10.1074/jbc.M116.762849.
67. Dickey, S. W., R. P. Baker, …, S. Urban. 2013. Proteolysis inside the membrane is a rate-governed reaction not driven by substrate affinity. *Cell*. 155:1270–1281. https://doi.org/10.1016/j.cell.2013.10.053.
68. Wang, Y., and Y. Ha. 2007. Open-cap conformation of intramembrane protease GlpG. *Proc. Natl. Acad. Sci. USA*. 104:2098–2102. https://doi.org/10.1073/pnas.0611080104.
69. Meleard, P., C. Gerbeaud, …, P. Bothorel. 1997. Bending elasticities of model membranes: influences of temperature and sterol content. *Biophys. J.* 72:2616–2629. https://doi.org/10.1016/S0006-3495(97)78905-7.
70. Bochicchio, D., and L. Monticelli. 2016. The membrane bending modulus in experiments and simulations. *In* Advances in Biomembranes and Lipid Self-Assembly. A. Iglic, C. V. Kulkarni, and M. Rappolt, eds, pp. 117–143.
71. Doktorova, M., D. Harries, and G. Khelashvili. 2017. Determination of bending rigidity and tilt modulus of lipid membranes from real-space fluctuation analysis of molecular dynamics simulations. *Phys. Chem. Chem. Phys.* 19:16806–16818. https://doi.org/10.1039/c7cp01921a.
72. Thewalt, J. L., and M. Bloom. 1992. Phosphatidylcholine: cholesterol phase diagrams. *Biophys. J.* 63:1176–1181. https://doi.org/10.1016/S0006-3495(92)81681-8.
73. Hung, W. C., M. T. Lee, …, H. W. Huang. 2007. The condensing effect of cholesterol in lipid bilayers. *Biophys. J.* 92:3960–3967. https://doi.org/10.1529/biophysj.106.099234.
74. Beverley, K. M., and I. Levitan. 2024. Cholesterol regulation of mechanosensitive ion channels. *Front. Cell Dev. Biol.* 12:1352259. https://doi.org/10.3389/fcell.2024.1352259.
75. Baccouch, R., E. Rascol, …, I. D. Alves. 2022. The role of the lipid environment in the activity of G protein-coupled receptors. *Biophys. Chem.* 285:106794. https://doi.org/10.1016/j.bpc.2022.106794.
76. Vogel, A., C. P. Katzka, …, D. Huster. 2005. Lipid modifications of a ras peptide exhibit altered packing and mobility versus host membrane as detected by ^{2}H solid-state NMR. *J. Am. Chem. Soc.* 127:12263–12272. https://doi.org/10.1021/ja051856c.
77. Arutyunova, E., P. Panwar, …, M. J. Lemieux. 2014. Allosteric regulation of rhomboid intramembrane proteolysis. *EMBO J.* 33:1869–1881. https://doi.org/10.15252/embj.201488149.
78. Skerle, J., J. Humpolickova, …, K. Strisovsky. 2020. Membrane protein dimerization in cell-derived lipid membranes measured by FRET with MC simulations. *Biophys. J.* 118:1861–1875. https://doi.org/10.1016/j.bpj.2020.03.011.
79. Leftin, A., T. R. Molugu, …, M. F. Brown. 2014. Area per lipid and cholesterol interactions in membranes from separated local-field ^{13}C NMR spectroscopy. *Biophys. J.* 107:2274–2286. https://doi.org/10.1016/j.bpj.2014.07.044.
80. Bruning, B. A., S. Prevost, …, T. Hellweg. 2014. Bilayer undulation dynamics in unilamellar phospholipid vesicles: effect of temperature, cholesterol and trehalose. *Biochim. Biophys. Acta*. 1838:2412–2419. https://doi.org/10.1016/j.bbamem.2014.06.006.
81. Schmidt, M. L., and J. H. Davis. 2017. Liquid Disordered-Liquid Ordered Phase Coexistence in Lipid/Cholesterol Mixtures: A Deuterium 2D NMR Exchange Study. *Langmuir*. 33:1881–1890. https://doi.org/10.1021/acs.langmuir.6b02834.

82. Martinez, G. V., E. M. Dykstra, …, M. F. Brown. 2004. Lanosterol and cholesterol-induced variations in bilayer elasticity probed by ^{2}H NMR relaxation. *Langmuir.* 20:1043–1046. https://doi.org/10.1021/la036063n.

83. Marquardt, D., F. A. Heberle, …, J. Katsaras. 2016. Lipid bilayer thickness determines cholesterol's location in model membranes. *Soft Matter.* 12:9417–9428. https://doi.org/10.1039/c6sm01777k.

84. Peruzzi, J. A., J. Steinkühler, …, N. P. Kamat. 2024. Hydrophobic mismatch drives self-organization of designer proteins into synthetic membranes. *Nat. Commun.* 15:3162. https://doi.org/10.1038/s41467-024-47163-1.

85. Greenblatt, E. J., J. A. Olzmann, and R. R. Kopito. 2011. Derlin-1 is a rhomboid pseudoprotease required for the dislocation of mutant alpha-1 antitrypsin from the endoplasmic reticulum. *Nat. Struct. Mol. Biol.* 18:1147–1152. https://doi.org/10.1038/nsmb.2111.

86. Baker, R. P., and S. Urban. 2012. Architectural and thermodynamic principles underlying intramembrane protease function. *Nat. Chem. Biol.* 8:759–768. https://doi.org/10.1038/nchembio.1021.

87. Neal, S., P. A. Jaeger, …, R. Y. Hampton. 2018. The Dfm1 Derlin is required for ERAD retrotranslocation of integral membrane proteins. *Mol. Cell.* 69:915. https://doi.org/10.1016/j.molcel.2018.02.014.

88. Bock, J., N. Kühnle, …, M. K. Lemberg. 2022. Rhomboid protease RHBDL4 promotes retrotranslocation of aggregation-prone proteins for degradation. *Cell Rep.* 40:111175. https://doi.org/10.1016/j.celrep.2022.111175.

89. Paschkowsky, S., S. J. Recinto, …, L. M. Munter. 2018. Membrane cholesterol as regulator of human rhomboid protease RHBDL4. *J. Biol. Chem.* 293:15556–15568. https://doi.org/10.1074/jbc.RA118.002640.

90. Marsh, D. 2009. Cholesterol-induced fluid membrane domains: a compendium of lipid-raft ternary phase diagrams. *Biochim. Biophys. Acta.* 1788:2114–2123. https://doi.org/10.1016/j.bbamem.2009.08.004.

91. Jakubik, J., and E. E. El-Fakahany. 2021. Allosteric modulation of GPCRs of class A by cholesterol. *Int. J. Mol. Sci.* 22:1953. https://doi.org/10.3390/ijms22041953.

92. Zakany, F., T. Kovacs, …, Z. Varga. 2020. Direct and indirect cholesterol effects on membrane proteins with special focus on potassium channels. *Biochim. Biophys. Acta.* 1865:158706. https://doi.org/10.1016/j.bbalip.2020.158706.

93. Smith, A. A., A. Vogel, …, D. Huster. 2022. A method to construct the dynamic landscape of a bio-membrane with experiment and simulation. *Nat. Commun.* 13:108. https://doi.org/10.1038/s41467-021-27417-y.

Biophysical Journal, Volume *123*

Supplemental information

Evaluating the impact of the membrane thickness on the function of the intramembrane protease GlpG

Oskar Engberg, Anjana V. Mathath, Viola Döbel, Christian Frie, Marius K. Lemberg, Debashree Chakraborty, and Daniel Huster

Supporting Information

Evaluating the Impact of the Membrane Thickness on the Function of the Intramembrane Protease GlpG

Oskar Engberg,[1] Anjana V. Mathath,[2] Viola Döbel,[1] Christian Frie,[3] Marius K. Lemberg,[3] Debashree Chakraborty,[2] Daniel Huster[1,*]

[1]Institute for Medical Physics and Biophysics, University of Leipzig, Härtelstr. 16/18,

D-04107 Leipzig, Germany

[2] Biophysical and Computational Chemistry Laboratory, Department of Chemistry, National Institute of Technology Karnataka, Mangalore, 575025, Karnataka, India

[3]Center for Biochemistry and Cologne Excellence Cluster on Cellular Stress Responses in Aging-Associated Diseases (CECAD), University of Cologne, Joseph-Stelzmann-Str. 52, D-50931 Cologne, Germany

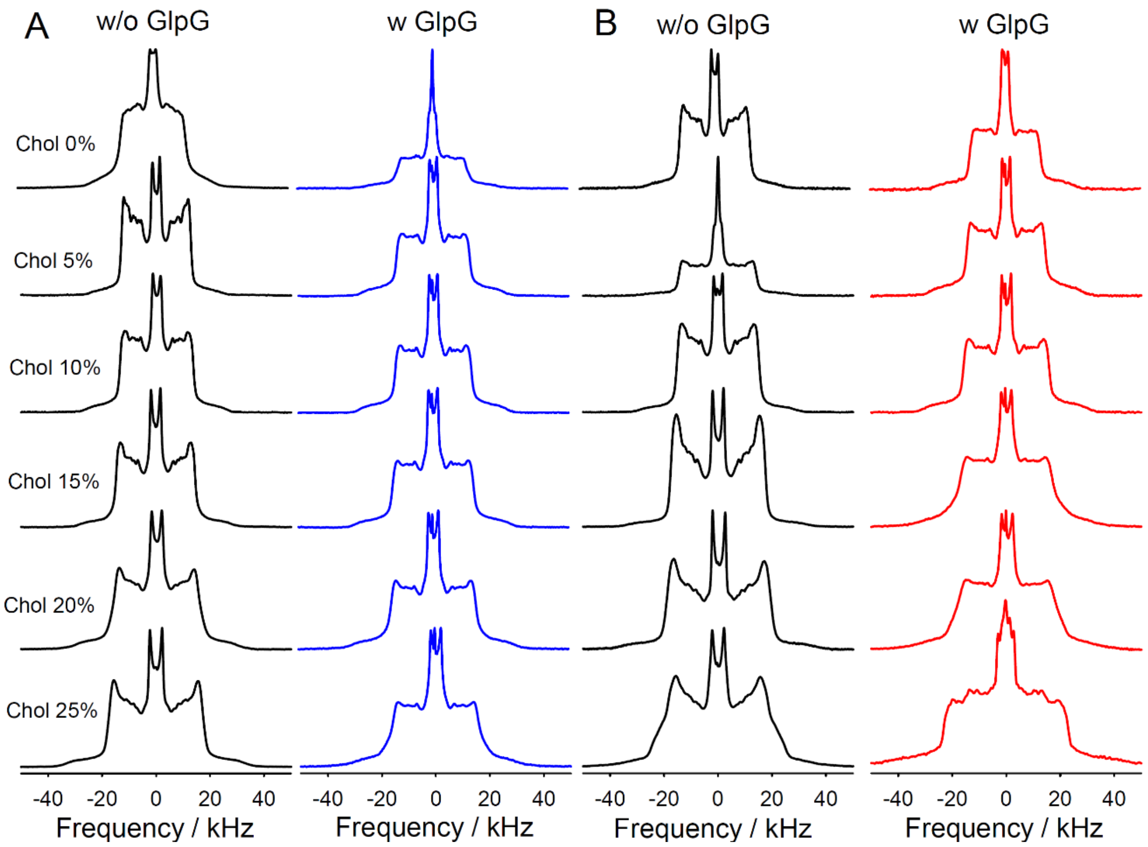

Figure S1. Static ^{2}H NMR spectra of DLPC-d_{46} and DMPC-d_{54} membranes with increasing amount of cholesterol in the presence and absence of GlpG. ^{2}H NMR spectra of DLPC-d_{46} in panel A) and DMPC-d_{54} in panel B. The indicated cholesterol concentrations correspond to the prepared concentrations. Measurements were carried out for MLVs hydrated with excess acetate buffer at pH 4 and a temperature of 37°C. The cholesterol-free spectra are reproduced from (17) with permission.

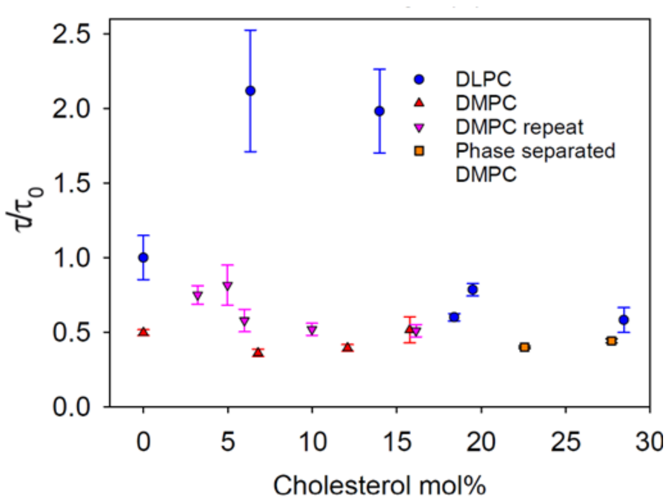

Figure S2. Effect of cholesterol content on GlpG-catalyzed LacYTM2 cleavage. The characteristic time constant τ that describes the cleavage reaction was calculated from the kinetic assay using FRET on the LacYTM2 model substrate and plotted against cholesterol concentration. The cholesterol concentration is measured from HPTLC lipid analysis. All τ values were normalized to the thinnest membrane DLPC with 0 mol% cholesterol (τ_0). All measurements were taken at 37°C.

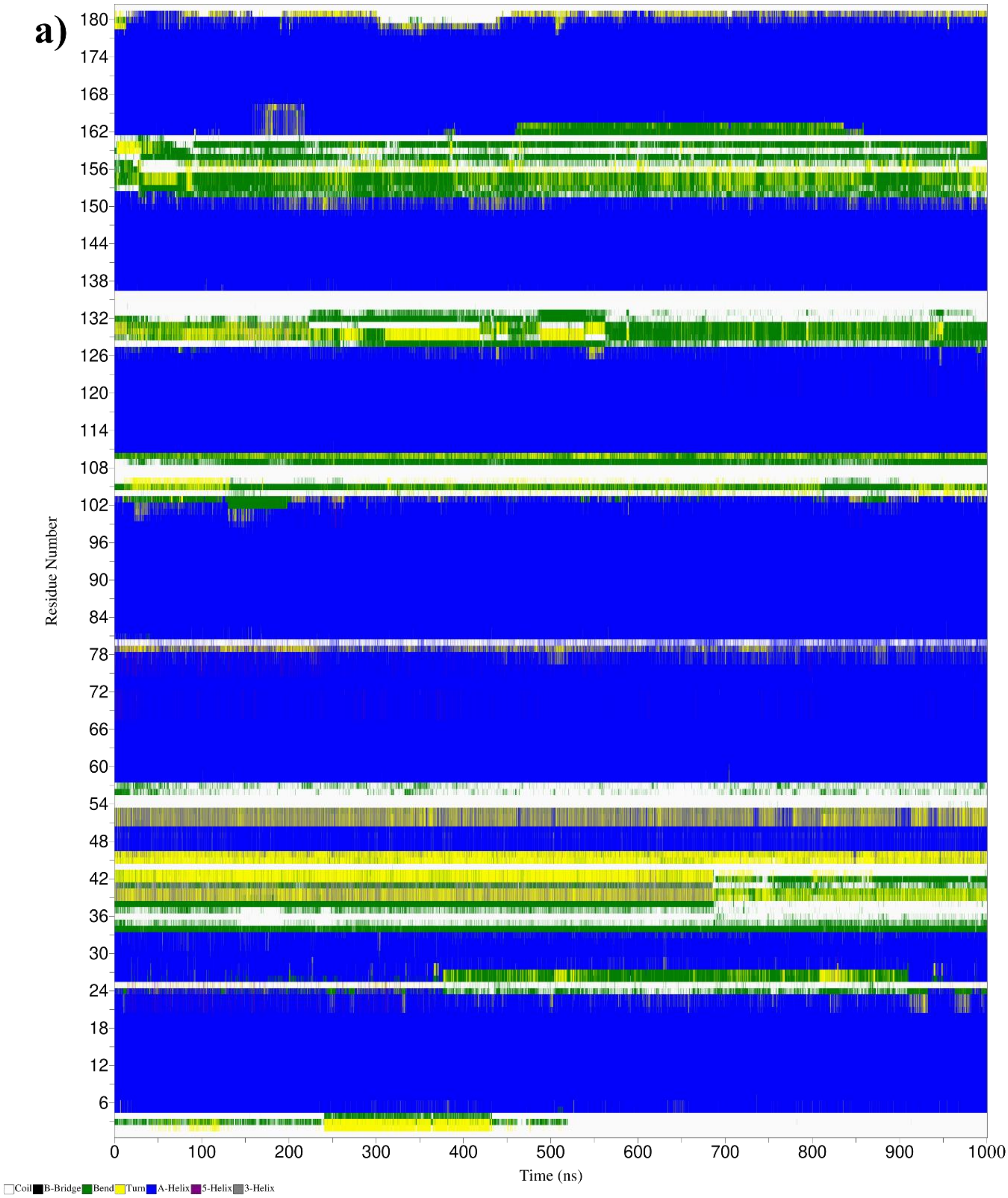
a)
Residue Number
180
174
168
162
156
150
144
138
132
126
120
114
108
102
96
90
84
78
72
66
60
54
48
42
36
30
24
18
12
6
0
100
200
300
400
500
600
700
800
900
1000
Time (ns)
Coil B-Bridge Bend Turn A-Helix 5-Helix 3-Helix

b)

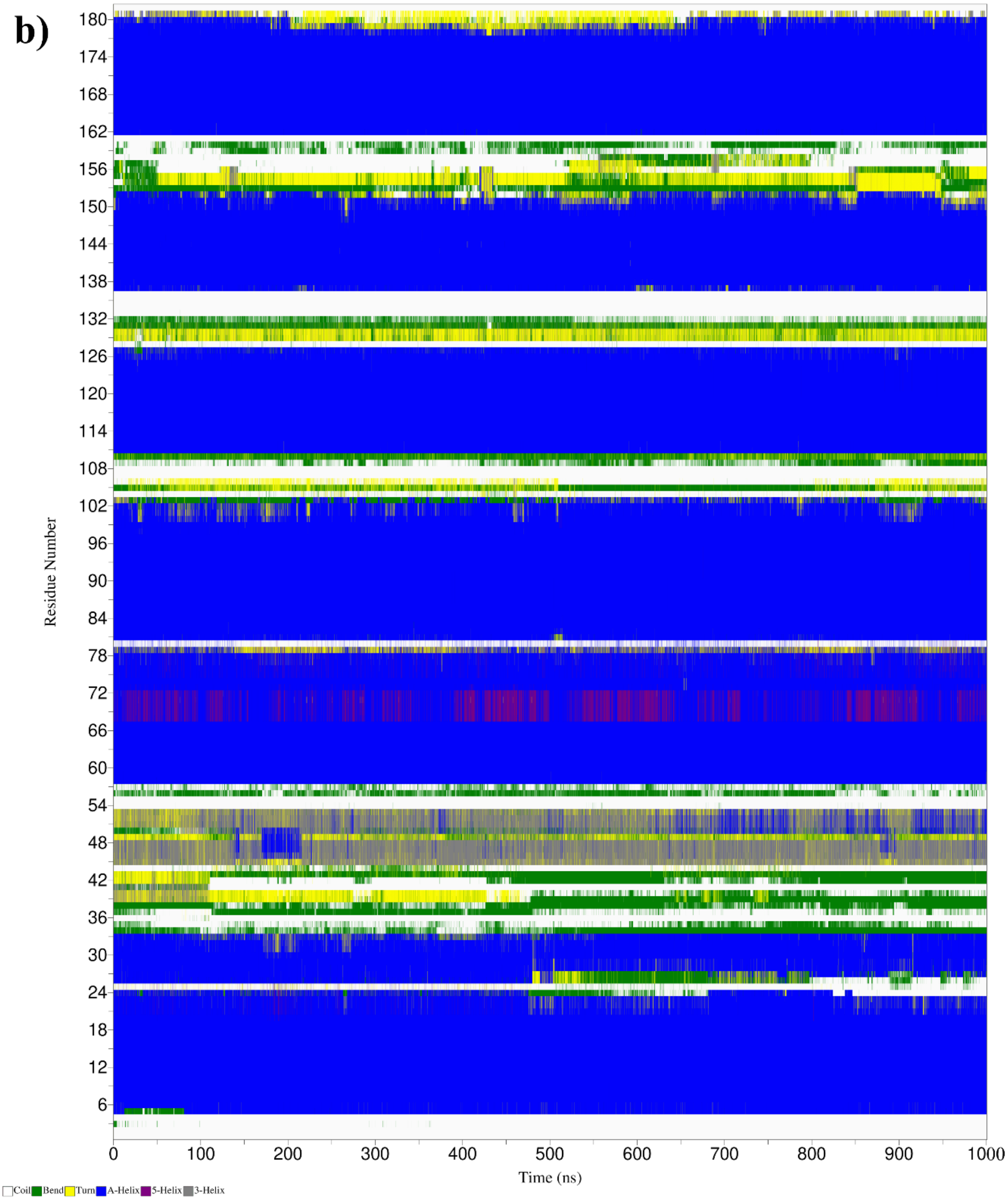

180
174
168
162
156
150
144
138
132
126
120
114
108
102
96
90
84
78
72
66
60
54
48
42
36
30
24
18
12
6
Residue Number
0
100
200
300
400
500
600
700
800
900
1000
Time (ns)
Coil Bend Turn A-Helix 5-Helix 3-Helix

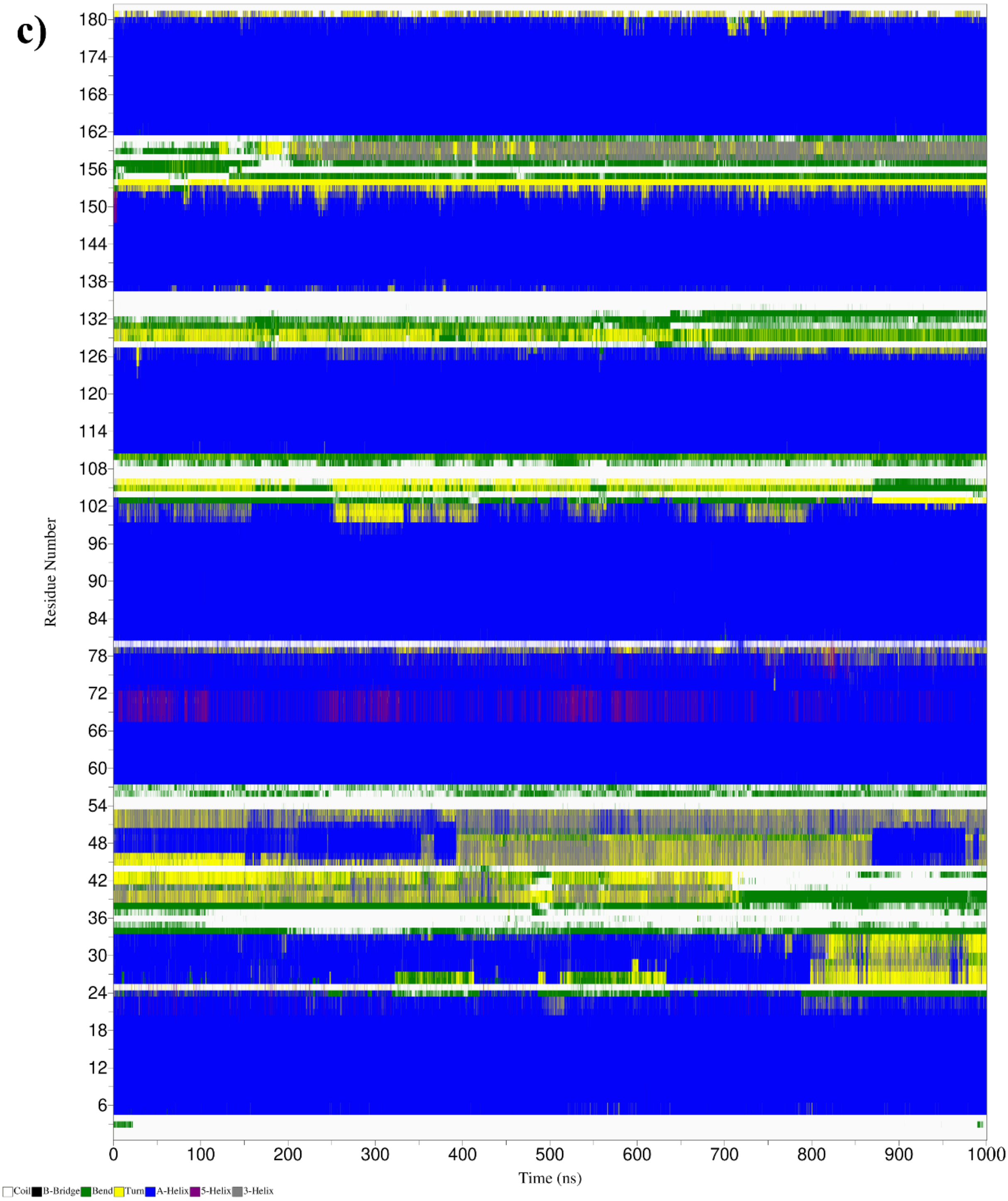
c)
Residue Number
180
174
168
162
156
150
144
138
132
126
120
114
108
102
96
90
84
78
72
66
60
54
48
42
36
30
24
18
12
6
0
100
200
300
400
500
600
700
800
900
1000
Time (ns)
Coil B-Bridge Bend Turn A-Helix 5-Helix 3-Helix

d)

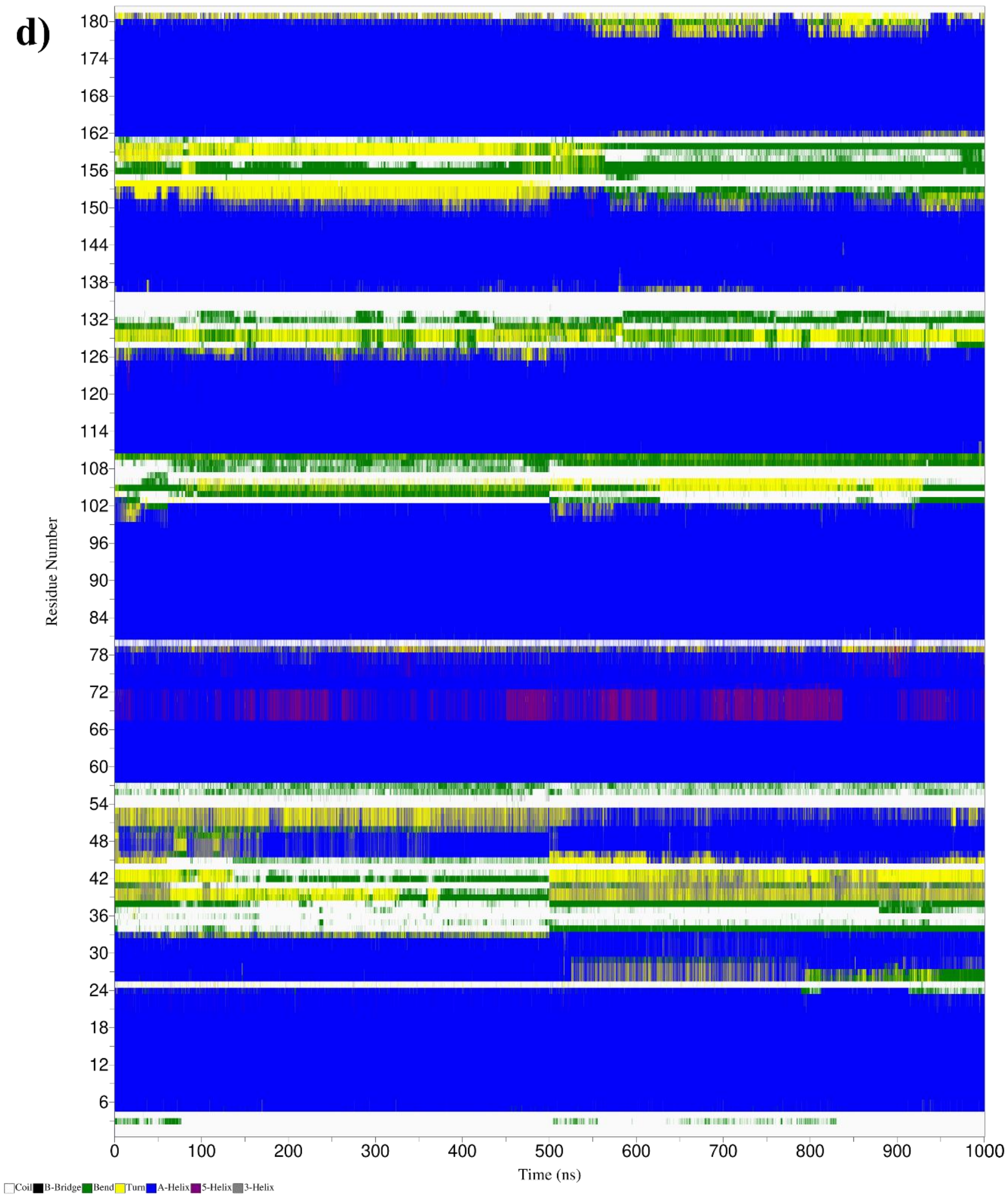

Residue Number
180
174
168
162
156
150
144
138
132
126
120
114
108
102
96
90
84
78
72
66
60
54
48
42
36
30
24
18
12
6
0
100
200
300
400
500
600
700
800
900
1000
Time (ns)
Coil
B-Bridge
Bend
Turn
A-Helix
5-Helix
3-Helix

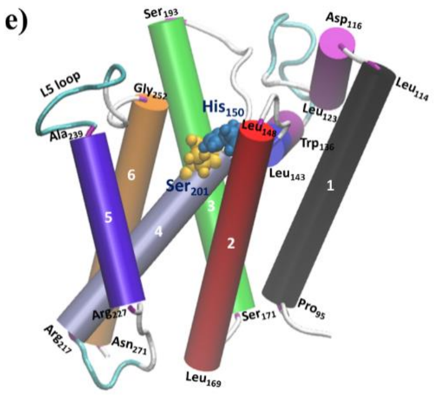

Figure S3. Secondary structure analysis of GlpG protein in DLPC systems at **a)** low cholesterol, **b)** high cholesterol, and in DMPC systems at **c)** low cholesterol and **d)** high cholesterol concentrations by dssp algorithm, **e**) Initial configuration of GlpG protein. GlpG contains six helices, which are labeled from 1 to 6, and contains 182 residues, starting from residue number 1 (Glu_{91}) to 182 (Ala_{272})*. The catalytic active center Ser_{201} (Residue no. 111) is on helix 4 (ice blue), and His_{150} (Residue no. 60) is on helix 2. Alterations for DLPC at low cholesterol are mainly found in regions 36-54, 68-72, and 153-161.

*Add 90 to each residue number in the secondary structure to match the literature residue number of respective amino acids in the sequence.

TM 1

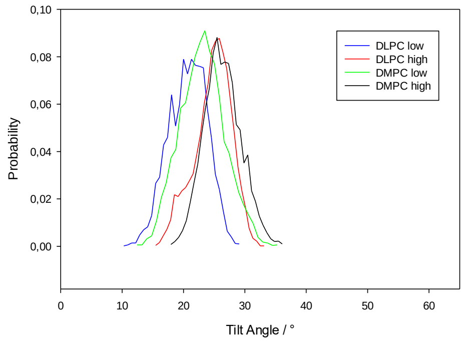

TM2

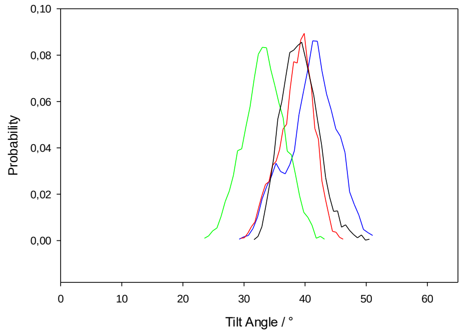

TM3

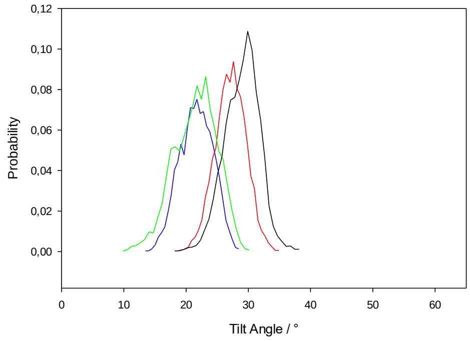

TM4

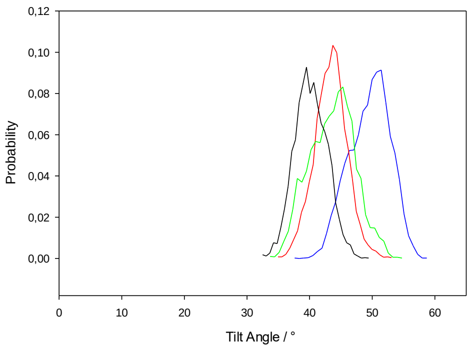

TM5

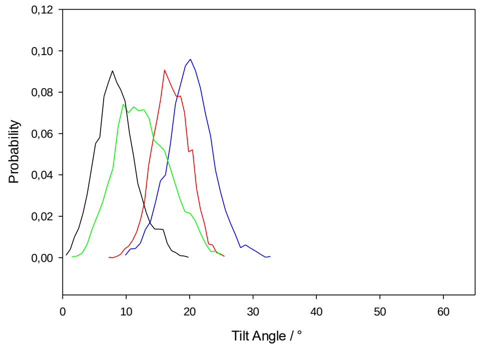

TM6

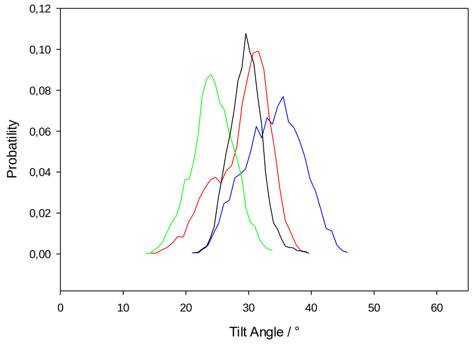

Figure S4. Histograms of the tilt angles of all transmembrane helices of GlpG in DLPC membranes at low (5%, blue curves) and high (30%, red curves) and in DMPC membranes at low (6%, green curves) and high (23%, black curves) cholesterol content calculated from the MD simulations.

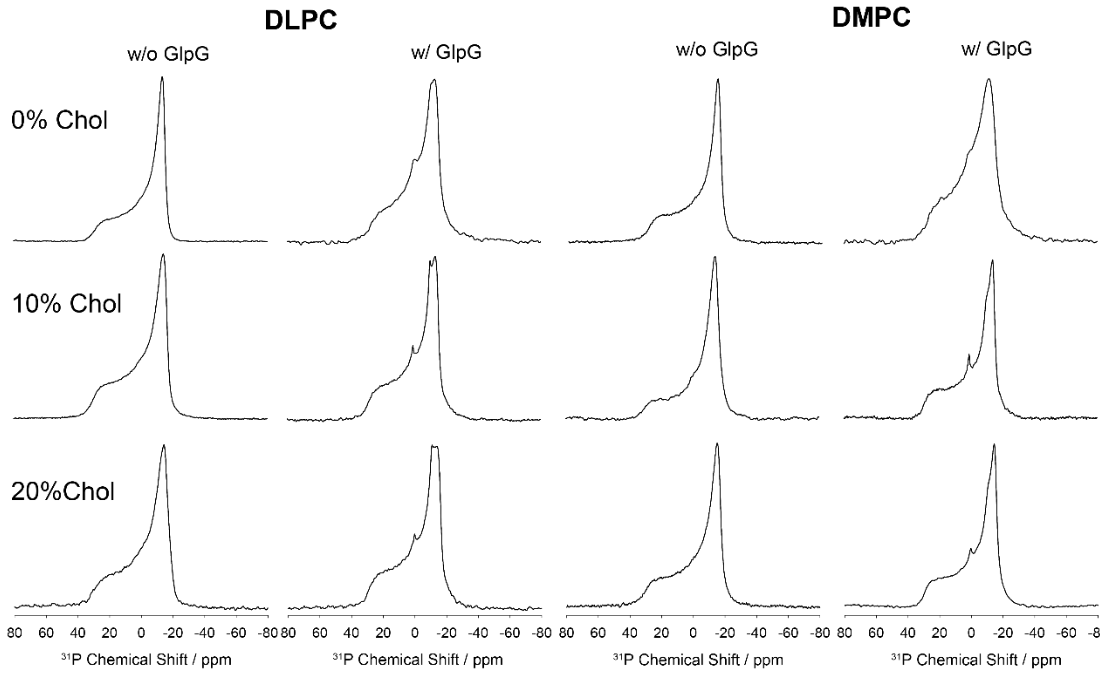

Figure S5. ^{31}P NMR spectra of all samples recorded from multilamellar vesicles in the absence and in the presence of GlpG at excess hydration and a temperature of 37°C at cholesterol concentrations as prepared.

DLPC at low cholesterol content (5%)

Annular cholesterol

Annular DLPC

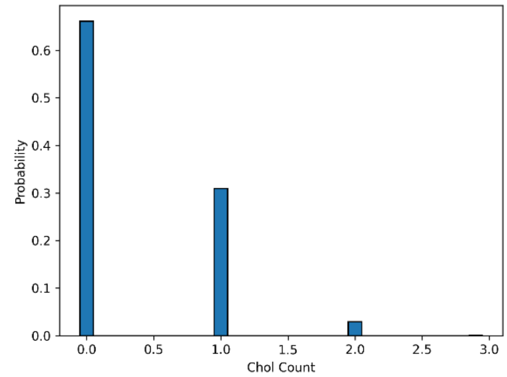

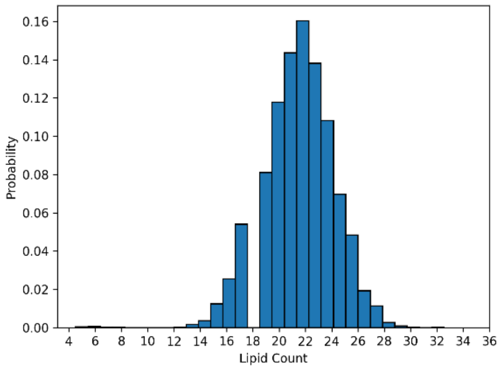

DLPC at high cholesterol content (30%)

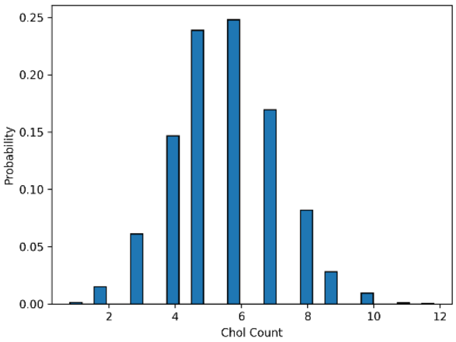

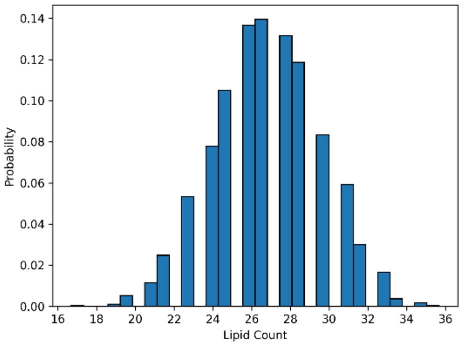

DMPC at low cholesterol content (6%)

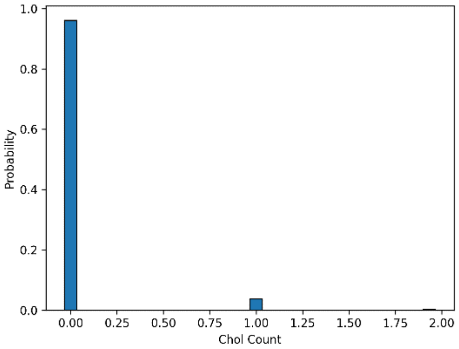

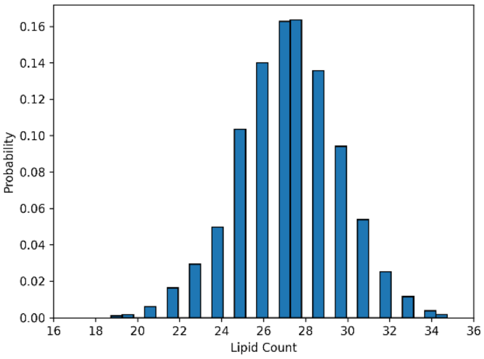

DMPC at high cholesterol content (23%)

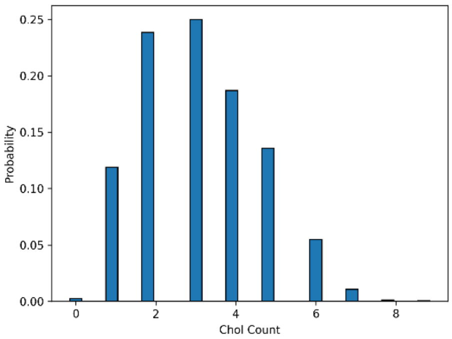

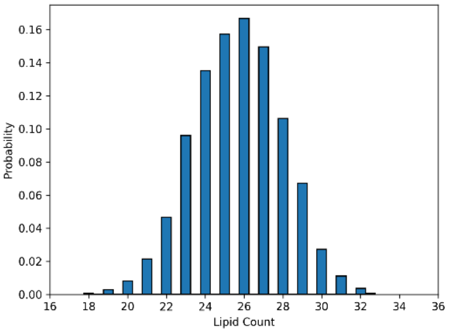

Figure S6. Histograms of the distribution of annular lipids (cholesterol vs. PC).

sn-2 chain order parameters for DLPC *sn*-2 chain order parameters for DMPC

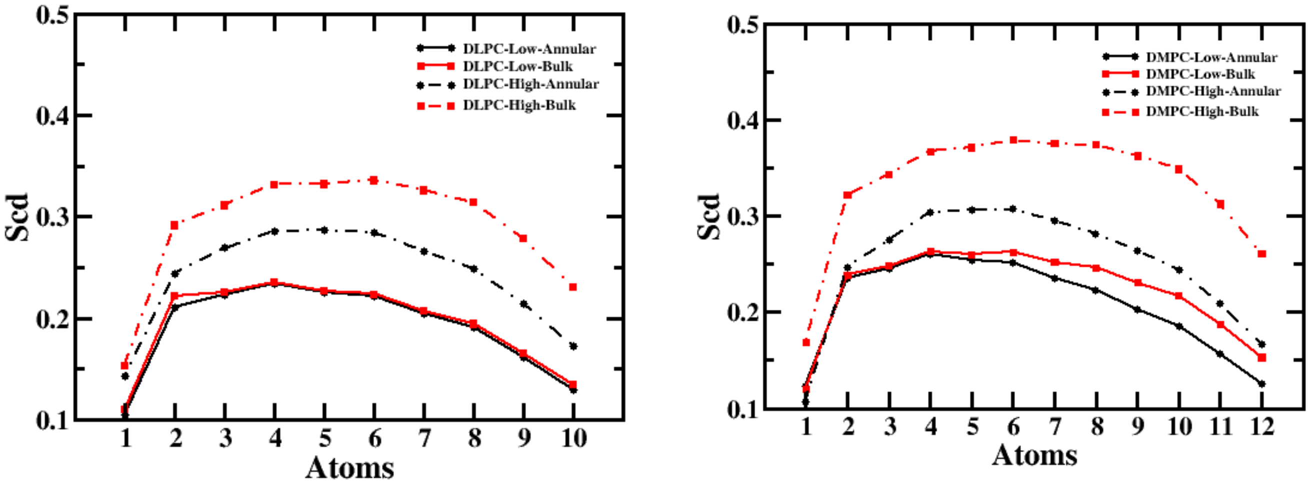

Figure S7. Lipid chain order parameters for the *sn*-2 chain of DLPC and DMPC at low and high cholesterol content in the presence of GlpG for the annular and bulk lipids calculated from the MD trajectories.

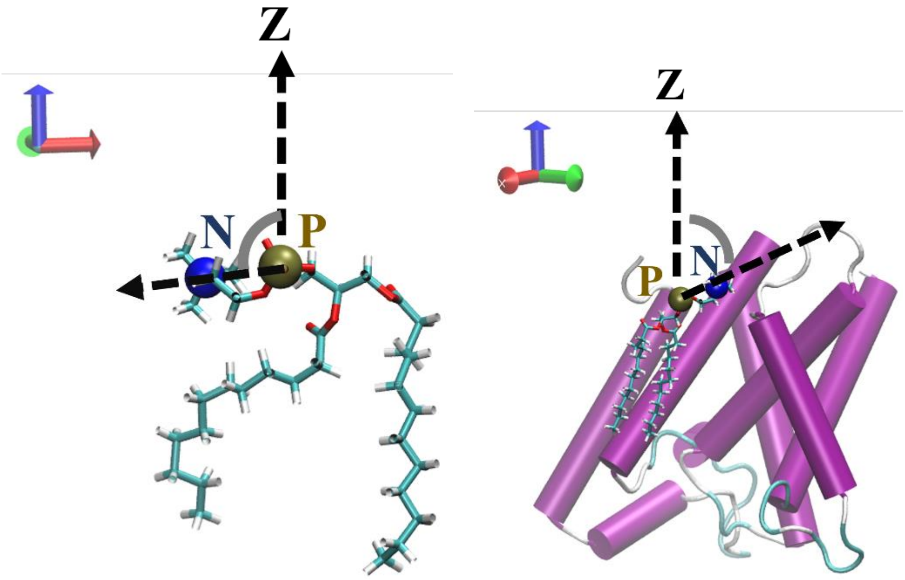

Figure S8. Pictorial representation of tilt angle computed between the lipid head group (P-N) vector and the bilayer normal for the bulk (left) and the annular lipids (right).

Table S1. Cholesterol concentration, Average order parameters (S_{CD}), projected chain lengths (*Lc*), number of *gauche* defects per chain, and area per molecule for deuterated DLPC and DMPC in the presence and absence of different GlpG at pH 4 at 37°C. Values for cholesterol-free membranes are reproduced from (17) with permission. NA = not applicable.

Phospholipid	Measured chol mol%	GlpG	S_{CD}	Lc (Å)	L/P ratio	SEM for L/P ratios	# gauche defects	A/Å2
DLPC-d_{46}	0.0	-	0.150	9.7			4.31	65.8
DLPC-d_{46}/Chol	7 ± 0.2	-	0.162	10.0			3.64	63.8
DLPC-d_{46}/Chol	13.4 ± 1.6	-	0.172	10.2			3.41	62.2
DLPC-d_{46}/Chol	19.4 ± 0.6	-	0.191	10.7			2.98	59.5
DLPC-d_{46}/Chol	22.5 ± 0.6	-	0.210	11.1			2.61	57.3
DLPC-d_{46}/Chol	28.6 + 1.3	-	0.235	11.6			2.15	54.8
DLPC-d_{46}	0.0	+	0.160	9.90	118	7	3.70	64.2
DLPC-d_{46}/Chol	8.8 ± 0.2	+	0.170	10.2	38.7	0.9	3.45	62.5
DLPC-d_{46}/Chol	15.3 ± 1.6	+	0.179	10.4	47.3	2.3	3.24	61.1
DLPC-d_{46}/Chol	23.71 + 0.5	+	0.192	10.7	44.3	0.6	2.98	59.5
DLPC-d_{46}/Chol	26.9 ± 2.6	+	0.201	10.9	54.8	1.8	2.81	58.5
DLPC-d_{46}/Chol	30.9 ± 1	+	0.210	11.1	48.9	1.1	2.64	57.5
DMPC-d_{54}	0	-	0.165	11.7			4.37	63.4
DMPC-d_{54}/Chol	6.4 ± 0.4	-	0.191	12.4			3.71	59.7
DMPC-d_{54}/Chol	10.9 ± 0.9	-	0.194	12.5			3.65	59.4
DMPC-d_{54}/Chol	12.9 ± 0.67	-	0.232	13.4			2.79	55.2
DMPC-d_{54}/Chol	18.9 ± 0.85	-	0.256	13.9			2.34	53.2
DMPC-d_{54}/Chol	26.9 + 1.2	-	NA	NA			NA	NA
DMPC-d_{54}	0.0	+	0.165	11.7	55	2	4.37	63.4
DMPC-d_{54}/Chol	7.3 ± 0.2	+	0.184	12.245	125.9	5.7	3.88	60.6
DMPC-d_{54}/Chol	8.9 ± 1.6	+	0.204	12.747	84.7	4.3	3.42	58.2
DMPC-d_{54}/Chol	14.1 ± 0.5	+	0.218	13.077	49.1	1.3	3.12	56.7
DMPC-d_{54}/Chol	15.6 ± 2.6	+	0.245	13.691	104.5	3.4	2.56	54.2
DMPC-d_{54}/Chol	23.4 ± 1	+	NA	NA	66.5	4.1	NA	NA

Table S2. The detailed simulation setup of systems in presence (*w GlpG*) and absence (*w/o GlpG*) of protein at low and high cholesterol (Chol) concentrations.

Systems	P/L ratio	% Chol	No. of Chol	No. of Phospholipids	No. of NaCl	No. of Water
**DLPC-Low** *(w/ GlpG)*	1:125	5	6	119	11	3823
**DLPC-Medium** *(w/ GlpG)*	1:125	16	20	105	10	3678
**DLPC-High** *(w/ GlpG)*	1:125	30	37	88	10	3518
**DMPC-Low** *(w/ GlpG)*	1:125	6	8	117	9	3436
**DMPC-Medium** *(w/ GlpG)*	1:125	15	19	106	9	3545
**DMPC-High** *(w/ GlpG)*	1:125	23	29	96	9	3460
**DLPC-Low** *(w/o GlpG)*	0:125	5	6	119	9	2375
**DLPC-High** *(w/o GlpG)*	0:125	30	36	89	5	2127
**DMPC-Low** *(w/o GlpG)*	0:125	6	8	117	5	2212
**DMPC-High** *(w/o GlpG)*	0:125	23	29	96	4	2108

Table S3. Analysis of bulk vs. annular lipids in the different system based on the histograms shown in Fig. S6 from the MD simulations.

System	Bulk lipids		Annular lipids	
	Cholesterol/phospholipid	Cholesterol content	Cholesterol/phospholipid	Cholesterol content
DLPC low	6/97	5.8%	0/22	0%
DLPC intermediate	19/78	19.6%	1/27	3.6%
DLPC high	31/61	33.7%	6/27	18.2%
DMPC low	8/90	8.2 %	0/27	0%
DMPC intermediate	17/80	17.5%	2/26	7.1%
DMPC high	26/70	27.1%	3/26	10.3%

Table S4: Cholesterol concentration, normalized τ-values, lipid/protein ratio (L/P) and substrate/protein ratio (S/P) of DLPC and DMPC in the presence of GlpG. NA = not applicable.

Phospholipid	Measured chol mol%	Normalized τ	SEM for normalized τ	Lc (Å)	L/P ratio	SEM for L/P ratio	S/P ratio
**DLPC**	0.0	1	0.15	9.9	82.7	5.7	0.55
**DLPC**	6.3 ± 0.4	2.12	0.41	10.1	92.3	4.7	0.56
**DLPC**	14.0 ± 0.5	1.98	0.28	10.4	109.4	6.2	0.62
**DLPC**	18.4 ± 2.1	0.60	0.02	10.5	69.5	4.5	0.62
**DLPC**	19.5 ± 0.3	0.79	0.04	10.6	74.9	4.6	0.61
**DLPC**	28.4 ± 1.9	0.58	0.08	10.9	74.7	3.8	0.69
**DMPC**	0.0	0.50	0.02	11.6	123.7	5.1	0.69
**DMPC**	6.8 ± 0.2	0.36	0.02	12.4	103.9	23.1	0.44
**DMPC**	12.1 ± 1.2	0.40	0.02	13.0	86.5	10.0	0.41
**DMPC**	15.8 ± 1.0	0.52	0.09	13.5	128.4	25.5	0.52
**DMPC**	22.6 ± 1.4	0.40	0.01	NA	98.9	5.5	0.60
**DMPC**	27.7 ± 1.7	0.44	0.01	NA	83.5	8.7	0.65
**DMPC repeat**	3.2 ± 0.1	0.82	0.13	12.0	49.4	6.3	1.18
**DMPC repeat**	5 ± 0.1	0.58	0.08	12.2	49.4	6.8	1.17
**DMPC repeat**	5.0 ± 0.4	0.52	0.04	12.3	56.7	3.0	1.19
**DMPC repeat**	10.0 ± 0.8	0.51	0.04	12.8	43.1	8.3	1.20
**DMPC repeat**	16.1 ± 0.5	0.69	0.06	13.5	38.4	12.2	0.92
